# Microarray-based resequencing of multiple *Bacillus anthracis *isolates

**DOI:** 10.1186/gb-2004-6-1-r10

**Published:** 2004-12-17

**Authors:** Michael E Zwick, Farrell Mcafee, David J Cutler, Timothy D Read, Jacques Ravel, Gregory R Bowman, Darrell R Galloway, Alfred Mateczun

**Affiliations:** 1 Biological Defense Research Directorate, Naval Medical Research Center, 503 Robert Grant Avenue, Silver Spring, MD 20910, USA; 2 Department of Human Genetics, Emory University School of Medicine, Atlanta, GA 30322, USA; 3 McKusick-Nathans Institute of Genetic Medicine, Johns Hopkins University School of Medicine, 733 North Broadway, Baltimore, MD 21205, USA; 4 The Institute for Genomic Research, 9712 Medical Center Drive, Rockville, MD 20850, USA

## Abstract

We used custom-designed resequencing arrays to generate 3.1 Mb of genomic sequence from a panel of 56 *Bacillus anthracis *strains. Sequence quality was shown to be very high by replication (discrepancy rate of 7.4 × 10^-7^) and by comparison to independently generated shotgun sequence (discrepancy rate < 2.5 × 10^-6^). Population genomics studies of microbial pathogens using rapid resequencing technologies such as resequencing arrays are critical for recognizing newly emerging or genetically engineered strains.

## Abstract 2

Custom-designed resequencing arrays were used to generate 3.1 Mb of genomic sequence from a panel of 56 *Bacillus anthracis *strains. Sequence quality was shown to be very high by replication and by comparison to independently generated shotgun sequence

## Background

Population genomics, the study of genome-wide patterns of genetic variation in a large number of organisms, is emerging as a vigorous new field of study [1-3]. Rapid, accurate and inexpensive resequencing could enable a variety of potential applications and studies. For the biowarfare (BW) pathogen, *Bacillus anthracis*, genomic sequences from multiple strains and non-pathogenic close relatives could aid studies that definitively identify *B. anthracis *in environmental and clinical samples, determine forensic attribution and phylogenetic relationships of strains, and uncover the genetic basis of phenotypic variation in traits such as mammalian virulence. Moreover, first recognizing the presence of a novel pathogen, and then attempting the difficult task of discerning between novel naturally occurring pathogenic organisms (for instance *Bacillus cereus *G9241 [4]) and artificially enhanced bacterial pathogens, requires a thorough knowledge of extant patterns and levels of genetic variation in natural populations. Unusual patterns of genetic variation may serve as evidence aiding the detection of these unusual types of pathogens.

The current technological model for genome sequencing employs high-throughput shotgun sequencing at large centers. This highly successful enterprise has completed about 200 bacterial genomes with more than 500 ongoing as of July 2004 [5]. The genome sequences of the *B. anthracis *Ames chromosome (5.2 Mb, NC_003997) and plasmids pXO1 (181.6 kilobases (kb), NC_001496) and pXO2 (96.2 kb, NC_002146) have been determined [6-8], as have the genomes of three near neighbors, *B. cereus *ATCC 14579 [9], *B. cereus *ATCC 10987 [10] and *B. cereus *G9241 [4]. A strain of *B. anthracis *Ames strain isolated from a victim of the autumn 2001 bioterror attack in Florida was also sequenced to a high level of coverage using the random shotgun method and compared to the Ames sequence to identify 60 new markers that included single nucleotide polymorphisms (SNPs), inserted or deleted sequences, and tandem repeats [11]. The success of this effort has led to an extensive phylogeny-based whole-genome shotgun resequencing effort in *B. anthracis *(reported by [12]). Whole-genome shotgun studies are increasingly being used to explore variation among more closely related bacterial strains [13-15]. However, the relatively high costs of these efforts have limited the extent of their application.

Numerous molecular methods for genotyping *B. anthracis *and near neighbors of the *Bacillus cereus sensu lato *group [16] have been developed and successfully employed in a wide variety of studies. These include DNA sequence surveys from one or a few number of loci [17-21], repetitive element polymorphism-PCR [22,23] and amplified fragment length polymorphisms (AFLP) [24-27]. However, because of the relative paucity of genetic variation between isolates [28], the most effective method for subtyping *B. anthracis *has employed multiple locus variable number of tandem repeats analysis (MLVA) [29-31]. Similar to the mammalian short tandem repeat methodology, MLVA determines strain phylogenetic relationships based on a relatively few, highly variable genomic repeat regions. While being relatively rapid and inexpensive, a key limitiation of MLVA lies in its exclusive focus on loci with common alleles that are differentiated by size. Because of the relatively rapid mutational process generating variation at these loci, similarly sized markers may have different evolutionary origins.

Clearly, a method for rapid, inexpensive genome resequencing of bacterial strains would be of great benefit for genotyping, forensics and studies of the genetic basis of strain phenotypic variation. Developing DNA-based biodetection assays depends upon prior knowledge of patterns of genetic variation within and between bacterial species. It would be ideal to enable technologies that could combine the high information content of whole-genome resequencing of strains while also being rapid and inexpensive like MLVA, AFLP and multi-locus sequence typing (MLST). Furthermore, while conventional strain typing methodologies have focused on the utility of common variants, rare variants may prove to be especially informative for forensic applications.

High-density oligonucleotide resequencing microarrays are a highly parallel technology that can enable the rapid identification of DNA sequence variants with minimal laboratory effort and infrastructure [32,33]. Previous applications of microarrays on bacterial genomes [34,35] or small eukaryotic genomes like yeast [36,37], focused on methods that scanned specific genes or a genomic region for genetic variants. Initial high-throughput microarray applications in the human genome for SNP discovery [38-40] were successful, but also reported that between 12% and 45% of the detected variants were false. Subsequent experimental improvements and the development of the ABACUS algorithm/software package [32] significantly reduced SNP false-positive ascertainment, radically improved genotype calling and automatically assigned quality scores to each genotype call. These fundamental advances enabled rapid resequencing of 40 human genomic regions [32,41] and ABACUS is now the standard application for microarray-based resequencing.

Here we present the first microarray-based high-throughput resequencing of a large collection of *B. anthracis *isolates. Our study first reaffirms, and then directly demonstrates that the quality of microarray-generated DNA sequence data is directly comparable to that produced by conventional shotgun sequencing. We then estimate the levels of genetic variation in the annotated genomic regions we resequenced, characterize the frequency spectrum of DNA sequence variants we observe, and finally explore patterns of linkage disequilibrium and recombination among those variants. Because of the scalability and minimal effort associated with microarray-based resequencing, our work demonstrates the possibility of a rapid and cost-effective method of genome resequencing that could be applied to both environmental, and ultimately clinical specimens.

## Results

### Resequencing *B. anthracis *with microarrays

A panel of 56 *B. anthracis *strains from the Biological Defense Research Directorate's strain collection (see Additional data file 1) was resequenced using Affymetrix resequencing arrays (RAs) and base calls determined using the ABACUS software package [32]. Each RA was capable of resequencing 29,212 base-pairs (bp) or about 0.5% of the *B. anthracis *genome from a single isolate sample (see Additional data file 2). Long PCR sample preparation and chip processing was conducted for 118 RAs. Analysis of these 118 RAs with the ABACUS software package shows that 115 are successful (97.5%). Experimental failure occurs when less than 60% of the total possible bases fail to achieve quality scores exceeding the ABACUS user-defined threshold. For this study, the total threshold was set at 31 and a strand minimum of -2 [32], as determined from analysis of a replication experiment described below.

**Figure 1:**
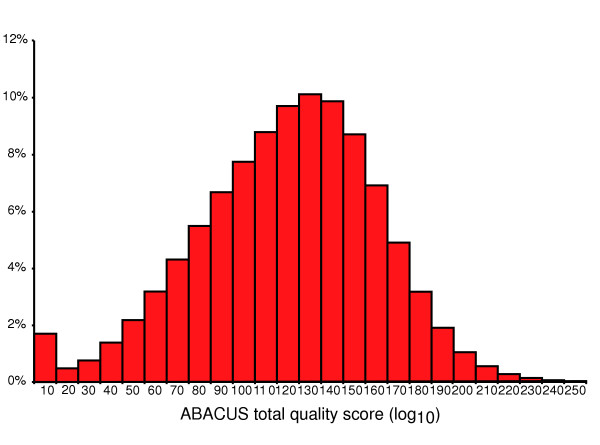
ABACUS quality scores for base calls in *B. anthracis*. A quality score measures the difference, in log_10 _units, between the likelihood support level for the best base-call model minus that for the second-best model [32]. Of the bases, 92.6% possess quality scores that exceeded the threshold (31) used for this study.

The 115 successful RAs call 92.6% of the possible bases (3,109,539 bp out of a total possible of 3,359,380 bp). Figure 1 shows the distribution of quality scores across all 3,359,380 base calls. Amplicon failure, typically arising from long PCR (LPCR) failure, accounts for 1.1% of the uncalled bases. The remaining base-calling failure (6.3%) consists of features on the RAs that fail to generate quality scores exceeding the experimental threshold.

Previous results demonstrated that base-calling failure was concentrated among RA oligonucleotide probes containing multiple purines. Purine-rich probes were observed to have lower hybridization intensities at identical positions across multiple RAs. Guanine-rich probes, in particular, showed the greatest reduction in hybridization intensity (see Figure 6 in [32]). Consequently, total quality scores at these sites frequently failed to exceed the quality-score threshold and they remained uncalled. To determine if probe sequence composition, specifically purine and guanine content, contributed to the 6.3% of bases not called, the sequence composition of the purine-rich oligonucleotide probes at 4,209 sites successfully called on all 115 RAs (484,035 total sites) was compared to that at the 886 sites that failed to be called on any RA (101,890 total sites). These failed sites account for 3.0% of the total base calling failure in the experiment. Uncalled sites are composed of oligonucleotide probes with a significantly higher purine composition (*P *< 10^-22^). A similar pattern is detected if we limit our analysis to guanine-rich probes (*P *< 10^-9^). This latter result is surprising given that the *B. anthracis *genomic sequences we examined have a low G+C content (~34%). Nevertheless, these analyses demonstrate that both purine-rich and guanine-rich oligonucleotide probes are significantly more likely to fail to generate quality scores exceeding the experimental threshold.

### Assessing microarray resequencing data quality

Building on the recognition of the importance of automated algorithms to assess data quality [42,43], we used two methods to assess the quality of microarray resequencing data [32]. The first consisted of a replicate experiment where 51 samples were independently hybridized on 102 RAs. A parameter search that optimized the percentage of called bases, while minimizing the number of discrepancies between replicates was then performed. A total of 1,489,812 bases could have been called in each replicate experiment. At the optimal parameter values (total threshold of 31, strand minimum of -2 see Cutler *et al. *[32]), 90.6% (1,349,178) of sites are called in both replicates. Other parameter values provide similar levels of base calling and discrepancy rates. The optimal parameter values are similar to those previously used by Cutler *et al. *[32]. Of the bases called in both replicates, 1,349,177 are called identically. Only one site is called differently. This corresponds to a replication discrepancy rate of 7.4 × 10^-7 ^(Table 1). If repeatability could be related to accuracy, then this level of repeatability would correspond to a phred score of at least 61 [42,43]. This calculation assumes that the discrepancy rate corresponds to a binomial error probability of *P*, where phred = -10 log_10_*P*. These replication levels and discrepancy rates are consistent with those previously reported [32], providing further evidence for the ability of RAs analyzed with ABACUS to produce highly replicable data.

While RA data is highly replicable, repeated systematic errors would not be detected in a replicate experiment. To obtain an independent estimate of RA sequence accuracy, we compared the sequence data from 30 RAs where the same *B. anthracis *strain had been sequenced using the random shotgun approach and deposited in GenBank (*B. anthracis*: strain Ames, NC_003997 [8], Vollum, NZ_AAEP00000000, 4 June 2004 update, strain Australia 94, NZ_AAES00000000, 7 June 2004 update, strain Kruger B NZ_AAEQ00000000, 7 June 2004 update (J Ravel, DA Rasko, MF Shumway, L Jiang, RZ Cer, NB Federova, M Wilson, S Stanley, S Decker, TD Read, *et al.*, unpublished work). In a comparison of 398,467 bp of RA- and shotgun-generated sequence, we observed 15 discrepancies occurring at six sites. This corresponds to a discrepancy rate of 3.8 × 10^-5^. If we make the conservative assumption that all discrepancies lie in the RA-generated sequence, this level of accuracy would correspond to a phred score of at least 44.

To determine if this conservative assumption is warranted, we examined in greater detail the nature of the RA/shotgun sequence discrepancies. Five of the discrepant sites, accounting for 10 discrepancies total (twofold RA replication at each site), were found in Kruger B strain sequences. The one remaining site, accounting for five discrepancies (fivefold RA replication at this site), was found in Vollum strain sequences. At all 15 discrepancies, the RA called a base identical to the Ames reference sequence [8], while the Kruger B/Vollum shotgun sequence called a new SNP. The fact that the shotgun sequence called a SNP at every discrepancy was surprising, leading us to examine more closely the level of shotgun coverage and assembly at each discrepant site. A comparison of the latest shotgun assembly of the Kruger B strain (J Ravel, *et al.*, unpublished work) with the RA Kruger B strain base calls agreed with the RA base calls. The latest Vollum shotgun assembly (J Ravel, *et al.*, unpublished work) still disagreed at the one site (five discrepancies total), but this discrepancy was based on a single shotgun sequencing read with a phred score of 7 at the discrepant base. Clearly, the shotgun coverage lacks sufficient depth at this site to make a reliable base call and it seems far more likely that the fivefold RA base call is correct. Hence, the RA sequence data has less than one discrepancy per 398,467 bases called, or a discrepancy rate of < 2.5 × 10^-6 ^(Table 1). This observed level of sequencing accuracy corresponds to a phred score of 56. These data demonstrate that our conservative assumption is not warranted. Resequencing array data quality from a single experiment matches, and in some cases perhaps exceeds, that obtained by multiple DNA sequencing reads using conventional DNA sequencing technologies [42,43].

### Patterns and levels of genetic variation in *B. anthracis*

We identify 37 SNPs among 56 *B. anthracis *strains. The SNP location, base-call, and position relative to the respective GenBank reference sequences [6-8] are contained in Additional data file 3. Twenty-four of the 37 SNPs, including two singletons, were independently confirmed in identical strains where whole-genome random shotgun sequence was available (A0039, A4088 and A0442 in Additional data file 1 (J Ravel, *et al.*, unpublished work)). Of the remaining 13 SNPs not independently verified by The Institute of Genomic Research (TIGR), 11 were seen only once in our collection of strains and two SNPs were seen three times.

Population genetic inference typically assumes that study samples are selected without prior knowledge of their patterns of genetic variation. For this study, we selected diverse strains from widely distant geographic regions in an attempt to sample the full extent of genetic variation in *B. anthracis*. The number of SNPs identified, the amount of sequence generated and the nucleotide diversity [44] of the 56 strains is contained in Table 2. We performed analyses for sequences comprising the total dataset, for each genomic region separately, and for the total dataset with each resequenced base assigned into an annotated SNP class. We report three main findings. First, the total average level of DNA sequence variation in *B. anthracis *is very low. This finding is in agreement with previous studies [11,28]. This level of genetic variation is much lower than that seen in commonly studied bacterial species [14], roughly half of that observed in the human genome and 25-fold lower than that observed in *D. melanogaster *[38,39,45-48]. Second, the *B. anthracis *chromosome appears less variable than either the pXO1 or pXO2 plasmids, although this difference is not statistically significant. Third, the patterns of genetic variation by SNP class (see Table 2 and Additional data file 4) are similar to that seen in other well studied bacterial [14] and eukaryotic genomes [45]. Silent sites, those sites that when mutated do not alter the protein primary structure, are significantly more variable than are amino acid altering replacement sites (*P *= 0.0011). Intergenic regions are observed to have intermediate levels of genetic variation, whereas replacement sites, those sites that when mutated alter the protein primary structure, are the least variable. Replacement sites are marginally significantly less variable than intergenic sites (*P *= 0.039) whereas silent sites are not significantly more variable than intergenic sites (*P *= 0.22).

**Figure 2:**
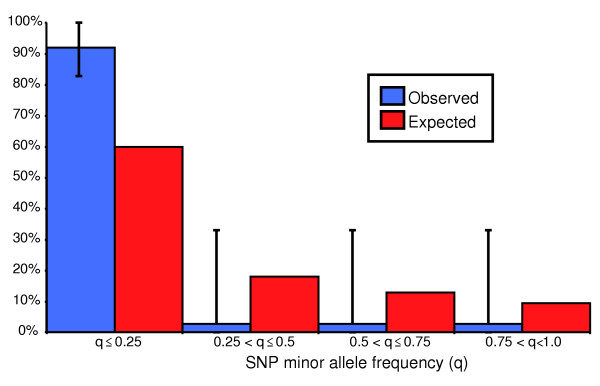
*B. anthracis *SNP frequency spectrum. An excess of rare SNPs are observed in our sample. Ninety-two percent of the SNPs that we discovered have a minor allele frequency less than or equal to 0.25. This finding (92%) is significantly different from the neutral theory expectation (60%). This excess can arise as a consequence of rapid, population expansion from a small founder population and/or the action of natural selection.

The neutral theory of molecular evolution predicts a characteristic frequency spectrum of SNPs, or segregating sites, for populations at equilibrium [49]. Deviations from this expected distribution are observed when an experimental population sample contains an excess of low frequency, rare SNPs, or an excess of high frequency, common SNPs, relative to the neutral expectation. These deviations can arise as a consequence of demographic history and/or the action of natural selection [50]. Figure 2 compares the observed and expected percent of SNPs in four allele-frequency classes. The data suggest an observed excess of rare SNPs as compared to that expected under the neutral theory. For example, while the neutral theory predicts that approximately 60% of SNPs should have minor allele frequencies less than or equal to 0.25, we observe that more that 92% of the *B. anthracis *SNPs we discovered have minor allele frequencies that fall into this class, a statistically significant difference (Figure 2).

We used the Tajima's D statistic [50] to further assess this pattern for the entire dataset, for SNPs from each genomic region and for each SNP class (Table 2). Tajima's D is a summary statistic for the site (or SNP) frequency spectrum, whose value is negative when there is an excess of rare variants and positive when there is an excess of common variants, relative to the neutral expectation. The test statistic is calculated from two different estimates of levels of genetic variation, the number of segregating sites [44] and the average number of nucleotide differences estimated from pairwise comparisons [50]. We observe that Tajima's D is negative for SNPs comprising the total dataset, each genomic region and each SNP class. While none of the individual test statistics is statistically significant, they collectively suggest an excess of rare variants in *B. anthracis*. If we scale our variation estimates drawn from the 0.5% resequenced in 56 *B. anthracis *genomes, we can estimate a range around the total number of SNPs that one would detect upon sequencing two random *B. anthracis *isolates, sampled in the same fashion as isolates in this study were chosen. Our results indicate that we should expect to find, on average, between 944 (standard deviation (SD) 454) [50] and 1,586 SNPs (SD 762) [44]. A substantial proportion of these SNPs, probably more than expected under the neutral theory, would be rare.

Using multiple sequence alignments of 17 genes from *B. anthracis *(NC_003997, Ames) and *B. cereus *(NC_004722, ATCC 14579 [9] and NC_003909, ATCC 10987 [10]) the patterns of genetic polymorphism and divergence at silent and replacement sites was assessed. The raw counts are presented in Table 3. It is striking that two *B. cereus *strains exhibit more polymorphism at silent and replacement sites than divergence from *B. anthracis*. This result confirms, at the DNA sequence level, previous results suggesting that the *B. cereus *species group is diverse and polyphyletic in origin. *B. anthracis *then appears to be a clonal lineage derived from, and nested within, a diverse species. In other words, the species names do not encompass or reflect the evolutionary history of the species [10,51,52].

### No evidence for recombination in *B. anthracis *chromosome

The 37 SNPs discovered on the *B. anthracis *chromosome and plasmids pXO1 and pXO2, possess in total, 636 pairs of sites where two alleles are observed. In principle, the alleles at each pair of sites could form four distinct haplotypes. Plasmid transfer between different *B. anthracis *strains would affect physically unlinked site pairs resulting in four distinct haplotypes. Homologous recombination or gene conversion between physically linked site pairs is also expected to produce all four haplotypes. The straightforward counting of the number of haplotypes that one detects in a large population sample, such as the one used in this study, is often referred to as the four-gamete test [53].

Among the 636 site pairs in our sample, we observe 26 pairs of sites with two haplotypes, 610 pairs of sites with three haplotypes, and no pairs of sites with four haplotypes. This striking result implies that the value of D', the standardized measure of linkage disequilibrium (LD) [54], is equal to 1, its maximum value, for all site pairs that we observe. Among the 137 site pairs where we could have detected statistically significant LD at *P *< 10^-3^, we observe that 52 site pairs exhibit statistically significant LD. Four of the six site pairs showing significant LD on the *B. anthracis *main chromosome are over 500 kb apart.

### Correlation of RA resequencing data with MLVA typing

Because of the low level of genetic variation in *B. anthracis *([28,29] and this study), determining the phylogenetic relationship among *B. anthracis *strains has proven difficult. Twenty-four *B. anthracis *strains characterized with a single fluorescent AFLP primer combination were reported to be monomorphic [27]. One recent MLST study sequenced seven housekeeping genes (approximately 3 kb total) in 5 *B. anthracis *strains and reported that the strains were monomorphic at the sites examined. Another recent MLST study sequenced seven genes (approximately 3 kb total) in 11 diverse *B. anthracis *strains finding three polymorphic nucleotides [55]. Neither the AFLP nor the MLST studies discover and genotype sufficient genetic variation to distinguish between *B. anthracis *strains.

**Figure 3:**
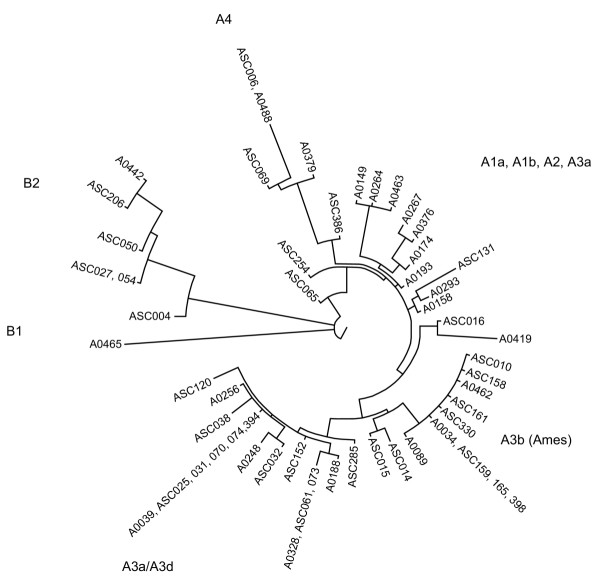
Radial tree showing inferred phylogenetic relationships of *B. anthracis *strains from this study. The 37 variable positions identified in this study were concatenated together to create artificial sequence types. Groups of strains with identical sequence types were A0488 and ASC006; A0039, ASC025, ASC031, ASC070, ASC074 and ASC394; ASC074 and ASC054; A0328, ASC061 and ASC073; A0034, ASC159, ASC165 and ASC398. A DNA distance matrix was created using DNADIST, plotted as a UPGMA tree using NEIGHBOR and the tree plotted using DRAWGRAM [56]. The B1 strain A0465 was used as an outgroup.

The most successful marker-based approach used to date, MLVA, determined the genotypes at eight VNTR loci in 426 *B. anthracis *isolates, enabling the construction of a phylogenetic tree of *B. anthracis *strains [29]. We sought to determine if our resequencing of 0.5% of each of 56 *B. anthracis *genomes is capable of confirming the major phylogenetic groupings determined by MLVA. To test this, we concatenated the 37 variant positions for all strains in this study, calculated a distance matrix using a simple Kimura substitution model, and generated an Unweighted Pair Group Method Arithmetic Mean (UPGMA) tree (see methods [56]; Figure 3). The strains group together in a manner broadly similar to that found by Keim *et al. *[29] with B strains forming an outgroup and most A strains being found together in the same subgroups (Figure 3). There are exceptions: one group in Figure 3 contains a mix of A3a, A1a, A1b and A2 strains. This anomaly is probably due to the relatively few SNPs that effectively distinguish these groups when only 0.5% of the genome is sampled. All *B. anthracis *Ames strains but ASC394 correctly cluster in an A3b group. *B. anthracis *ASC394 may be a case of an originally mistyped or mislabeled strain. Nevertheless, our data suggest that limited, random resequencing of 0.5% of the 56 *B. anthracis *genomes discovers and genotypes sufficient genetic variation to determine the major phylogenetic relationships among *B. anthracis *strains.

## Discussion

Population genomics requires the random sampling of genome-wide patterns of DNA sequence variation in a large number of organisms. Such studies require high-throughput, highly accurate, cost-effective resequencing technologies. While the conventional industrial-scale shotgun-sequencing model is clearly the best technology available for *de novo *generation of genomic sequence, it may not be the best approach for resequencing large numbers of strains. RAs, as originally applied for human genome resequencing [32], offer one competing technology that can rapidly produce very high-quality data with limited personnel and infrastructure requirements. Our application of RAs to resequence multiple genomic regions in the biowarfare pathogen, *Bacillus anthracis*, further supports this perspective.

Studies of DNA sequence variation are most informative when both rare and common variants are identified. While the limited ascertainment of selected common variants can be employed to identify broad evolutionary relationships among bacterial genomes, and in fact underlies most bacterial strain typing methodologies, the ultimate forensic application of resequencing lies in the ascertainment of rare, presumably newly arising variants, that may allow more precise determination of a strain's origin. Rare variants may be particularly informative since they are likely to be restricted to specific strains (substrains/isolates). Strain genotyping of common variants provides an incomplete description of genomic patterns of DNA sequence variation, while obtaining most or all of the genomic sequence from multiple strains allows a maximally informative analyses of DNA sequence variation, its function, and ultimately, the evolutionary history of the organisms. The ability to rapidly, accurately and inexpensively resequence entire bacterial genomes should also contribute to an understanding of a variety of important phenotypic traits in *B. anthracis *and other bacterial pathogens [57-62].

Our study demonstrates that microarray-based resequencing is technologically robust and generates highly replicable and accurate data when compared to alternative sequence technologies (Table 1). In this experiment, 115 RAs, or 97.5% of the total attempted, were processed successfully obtaining an average high-quality base-calling rate of 92.6%. Called bases are shown to be highly replicable (discrepancy rate of 7.4 × 10^-7^) and accurate when compared to conventional shotgun sequence (discrepancy rate of < 2.5 × 10^-6^). Clearly, RA-generated resequencing data from a single experiment is comparable, in terms of data quality, to DNA sequence generated from multiple shotgun reads by a DNA sequencing center. The major technical challenge facing RA-based resequencing is to increase overall call rates while not compromising data quality. Modifications of RA synthesis, experimental protocols and the ABACUS software algorithm could all contribute to improved base-calling rates. While it is possible to increase call rates while sacrificing data quality, there is a need to focus on generating very high-quality data at virtually all sites. If this is absent, the second-best outcome is to call all bases in an environment in which we understand the nature of probable errors. In diverse fields where RAs might be widely used as a first-stage screening tool, such as BW agent identification or human clinical testing, the imperative is to use highly sensitive technologies that minimize the false-negative rate. False-positive findings could be confirmed later in a second-stage screen with an alternative technology such as conventional dideoxy chain termination sequencing.

Microarray-based resequencing identifies and genotypes SNPs in a single experiment. No prior knowledge of the variability of a site is required - only a reference genomic sequence. Microarray design and applications are flexible. It is, however, important to note that the use of RAs in this study is not as a SNP typing technology. Thus, problems in interpreting the inferred phylogenetic relationships between strains that arise from SNP typing schema are avoided [63]. RA-based resequencing resembles MLST methodology used for bacterial strains [52,55,64]. MLST attempts to choose the most informative genomic regions to resequence, largely because of the costs associated and technological limitations in generating enough DNA sequence data on a large collection of variant strains. While a typical MLST approach might resequence between 3 and 4 kb, in organisms like *B. anthracis *that have low levels of genetic variation ([28,51,55] and this study), this amount of generated sequence is insufficient. Clearly, RAs, such as those used in this study that can resequence approximately 29 kb, could rapidly increase this amount and be used for MLST studies. Furthermore, manufacturing improvements that reduce RA feature sizes enable the resequencing of greater quantities of genomic sequence per microarray. Ongoing work at NMRC/BDRD is evaluating RAs that can resequence 300 kb per chip. At that RA feature density, when combined with whole-genome amplification protocols, a single technician in two days could resequence the entire *B. anthracis *genome on approximately 15 RAs.

Our data provides the first population genetic estimation of the levels and patterns of DNA sequence variation in *B. anthracis*. We report three main findings. First, among *B. anthracis *isolates sampled in the same fashion as in this study we would expect two randomly selected *B. anthracis *strains to differ, on average, at between 944 (SD 454) and 1,586 SNPs (SD 762). The variance surrounding these expectations is large, and any two isolates may differ from the expectation. Closely related, nonrandomly sampled isolates, such as those sequenced in [11], will have far fewer SNPs than that expected for samples drawn from a worldwide collection. Nevertheless, our data suggest that were it possible to rapidly resequence entire *B. anthracis *genomes, sufficient genetic variation is likely to be found to make very fine-level discrimination of strain collections. Resequencing offers the best chance to identify newly arising, rare, strain-specific variants that will discriminate between very closely related strains, since we expect identical genotypes at the known common genetic variants [11]. We also observe, that as seen in eukaryotic genomes [45], the amount of silent variation per site within genes is much higher than that seen at replacement sites. Intergenic regions are seen to have intermediate levels of polymorphism. This pattern is expected to arise if noncoding intergenic regions possess variants visible to natural selection. If SNPs in intergenic regions were purely neutral, then we would expect to see levels of polymorphism similar to that at silent sites, which are undoubtedly under less stringent selective forces.

Second, the neutral theory of molecular evolution predicts that in a population at equilibrium, a significant proportion of the observed genetic variation will consist of rare genetic variants [49]. We observe a significant excess of rare SNPs as compared to that expected under the neutral theory (Table 2). This pattern of variation classically has at least two possible causes. The first consists of a recent population expansion from a small founder population. The second consists of the action of natural selection on genetic variants [65-67]. Resequencing technologies will be of particular use in populations of organisms exhibiting this pattern of genetic variation.

Finally, we see no evidence for plasmid exchange or recombination altering the patterns of DNA sequence variation among *B. anthracis *strains in the regions that we resequenced. Some of the regions that we resequenced contain genes whose function influences *B. anthracis *pathogenicity or surrounded the bacterial origin of replication. In other bacterial species, these types of regions are the most likely to exhibit recombination [14]. The fact that we observe no evidence of plasmid exchange or recombination among physically linked markers in the regions that we resequenced, is striking.

The simplest interpretation of this observation is that the *B. anthracis *strains that we examined are ultimately derived from a single clonal ancestor and that the exchange of plasmids and recombination between strains during the course of their evolution is either very rare or nonexistent. While models of natural selection could also account for the patterns that we see, we think a simple demographic model of recent, rapid clonal expansion is parsimonious and best supported by our data. Hence, our findings suggest that *B. anthracis *populations consist of multiple closely related clones whose life histories prevent the opportunity for homologous recombination between different strains. We note, however, that while we resequenced 0.5% of the *B. anthracis *genome, including regions where we expected to detect recombination, further data collection from multiple genomic regions, or the entire genome, would allow a more thorough analysis of this pattern. Sequencing a larger percentage of the genome in a similar-sized or larger sample of isolates would provide greater power to detect rare recombination events. We are undertaking such a project to test the validity of our inference and to better determine if recombination is rare or absent among *B. anthracis *strains.

The absence of recombination in *B. anthracis*, a potential biowarfare agent, suggests a novel approach to identifying a newly arising or a genetically engineered strain. A recombination event could arise through rare natural genetic exchange or as a consequence of genetic engineering. Irrespective of the cause, the discovery of a *B. anthracis *strain possessing evidence of genetic recombination would warrant close examination and probably demand immediate further phenotypic and genomic characterization.

Taken together, the findings of a low number of differences between strains, a preponderance of rare variants, and an absence of recombination all point to a scenario where the current world population of *B. anthracis *has expanded recently from a single clone derived from, and nested within a diverse species, *B. cereus*. Other bacterial pathogens, such as the potential biowarfare agent *Yersinia pestis*, possess a similar recent pattern of rapid expansion [15]. However, the patterns of genetic variation in *Y. pestis *are quite different from that seen in *B. anthracis*, for instance in the much more active role of insertion sequences in *Yersinia*. We speculate that the *B. anthracis *history of clonal expansion could arise as a consequence of the life history of a highly pathogenic sporulating mammalian pathogen. Exploring the population biology of less virulent members of the *B. cereus *group could directly test this. These population genomics studies could determine if clonal clusters of *B. cereus *strains exhibit similar population dynamics and patterns of genetic variation, or whether the picture of *B. anthracis *emerging from studies such as this is as unusual as the level of pathogenicity of the species itself.

## Conclusions

Microarray-based resequencing can rapidly generate very high quality data, enabling population genomics studies in bacteria. We find no evidence for plasmid exchange or recombination altering the patterns of DNA sequence variation among *B. anthracis *strains in the regions that we resequenced The patterns of genetic variation in the *B. anthracis *regions resequenced are consistent with that expected for a bacterial species that has undergone a rapid, historically recent expansion from a single clone. Detecting plasmid exchange or recombination between *B. anthracis *genetic variants could act as an indicator of a newly emerging or genetically engineered strain.

## Materials and methods

### *B. anthracis *strains surveyed

We selected a geographically diverse panel of 56 *B. anthracis *strains from the Biological Defense Research Directorate collection (see Additional data file 1). Twenty-four of the strains originated from the Louisiana State University collection [29]. These have been typed by MLVA [29] and in order to sample diversity, we chose a group that had representatives of the A1a, A1b, A2, A3a, A3b, A3d, A4, B1 and B2 lineages. The remaining 35 strains originate from a UK collection and were chosen to represent geographical variation as well as unusual phenotypes such as gamma phage and penicillin resistance. Six of the UK strains were reisolates of the Ames strain [11], which allowed us to test the reproducibility of resequencing.

### Resequencing array design

Unique genomic sequences were identified using Miropeats [68] at the default thresholds from among the *B. anthracis *Ames chromosome (5.2 megabase-pair (Mb), NC_003997) and plasmids pXO1 (181.6 kb, NC_001496) and pXO2 (96.2 kb, NC_002146). The genomic regions that we resequenced included at least one gene of interest (pXO1: toxin lethal factor precursor *lef*, toxin moiety, protective antigen *pagA*; pXO2: encapsulation protein gene *CapC*; Ames chromosome: *vrrA*, DNA-directed RNA polymerase *rpoB*, *yfhp *protein), but also included many surrounding loci (see Additional data file 4 for complete listing). The total chip design consisted of 6,191 bp from pXO1, 6,725 bp from pXO2, and 16,584 bp from the Ames chromosome (total submitted sequence 29,500 bp). From these unique sequences, a single 20 × 25 μm RA design capable of resequencing 29,212 bp or 0.5% of the *B. anthracis *genome was fabricated by Affymetrix (see Additional data file 3). The final sequences submitted for RA design are contained in Additional data file 5.

### *B. anthracis *strain genomic DNA isolation

Five milliliters of brain heart infusion (BHI) was inoculated and grown 12-16 h at 37°C. One-ml aliquots of cells were centrifuged for 10 min at 5,000-7,500*g*. Pellets were resuspended in 720 μl enzymatic lysis buffer (20 mM Tris-Cl pH 8.0, 2 mM EDTA, 1.2% Triton X-100, 20 mg/ml lysozyme) and incubated at 37°C for 1 h. After incubation 100 ml of Proteinase K was added along with 800 ml of Qiagen buffer AL, and incubated at 70°C for an additional 30 min. Then, 800 ml of 100% ethanol was added and this was split onto four of the Qiagen DNAeasy tissue kit. The DNA was then washed and eluted according to the Qiagen protocol. After the DNA was eluted, it was passed through a 0.22 mm filter. Sterility was confirmed by plating 10% DNA preparation directly on SBA plates with a second 10% inoculated into a 5 ml broth culture. The plate and the broth were allowed to incubate for 7 days. Two hundred milliliters of the broth culture was subcultured onto SBA at day 4. If there was no growth on any of these cultures the DNA was considered sterile and removed from the BSL-3 lab for subsequence analyses.

### Sample preparation and RA hybridization

Genomic DNA was amplified using Long PCR (LPCR) protocols described in Cutler *et al*. [32]. The primers that amplified each RA fragment are shown in Additional data file 3. The primer sequences were:

ant8 AAAAAGACGAGATGCGTCAACATCCCGTCCCA,

ant9 TCAACTAAATCCGCACCTAGGGTTGCTGTAAG,

ant10 ATTACTTTGAGTGGTCCCGTCTTTATCCCCCT,

ant11 ACATTAGCAGGCAAGGACAGTGGTGTTGGAGA,

ant14 ATTCACGCTCTCCCACCCAGATATTCCTACAT,

ant15 GTCCTAATATCGGTGAGCAACGCAGGGTAGTT,

ant20 GAGAAGAACCCCTACTACACGCATTGATACTG,

ant21 TTTAGTAGCGAGGGTACAGGCGCGTTTATACC,

ant26 TGGAAGCAGGCTTCGTAAGTGTAGGCGACGTT,

ant27 GTTGCATGTTCGCTCCCATAAGTGCGCGGTTA,

ant 32 AATGGGTGTATAGGGGTGATCTGTTGTGATGG,

ant33 TCCATGTTCGGCCATCTGATTCCGTCACTACT.

Long PCR product concentration was determined by using Pico-Green (Molecular Probes, Inc.) with lambda DNA standards (Invitrogen). The LPCR products were then pooled, DNAse digested, biotin endlabelled and hybridized to individual RAs overnight following established protocols contained in [32]. Subsequent washes and stains were carried out as described in Cutler *et al. *[32] and were only washed and not antibody stained. RAs were scanned at 570 nm, with a pixel size of 3 μm/pixel averaged over two scans. Automated grid alignment and base calling was performed for the .DAT files on a Mac G5 computer with the ABACUS software suite.

### RA sequence determination

An ABACUS parameter search was employed to determine those parameters that called the maximal number of bases while minimizing discrepancies [32]. This total experiment consisted of 118 RAs, of which three failed (< 60% base calling). Of the remaining 115 RAs, 8 were used to sequence individual strains once. Of the remaining 107 RAs, 96 were used to replicate hybridize 48 *B. anthracis *strains, while the remaining 11 RAs were used as additional multiple replicates of these same strains. In total, sequence data was generated from 56 unique *B. anthracis *strains (see Additional data file 1 for strain listing). In order to obtain the most complete data possible, for those strains with replicate RA sequences, a single composite strain sequence was generated for subsequent population genetic analyses. The current version of ABACUS algorithm is not designed to detect insertion/deletion variation.

The effect of oligonucleotide probe composition was determined by choosing for each base, the probe with the most purines or the most guanines. The number of times that a given base was called was tabulated across all 115 successful RAs. The mean purine and guanine composition was determined for the classes that were called in all 115 RAs and uncalled in all 115 RAs. A Student's *t *test with unequal variances was used to test for difference in mean sequence composition (purines/guanines) between the always called and never called classes. The DNA sequence files for the 115 RAs and the original RA image files (.DAT files) are available from the authors and will be made available through the NCBI Trace Archive.

### Population genetic analyses

All population genetic analyses were calculated using the popgen_fasta2.0.c code (Cutler DJ, unpublished work) on the collection of 56 sets of *B. anthracis *fasta files. The fasta files were analyzed in total and separately for the main chromosome and plasmids pXO1 and pXO2. The identification of genes was taken from publicly available annotation contained in the relevant GenBank refseq files (*B. anthracis *str. Ames NC_003997; pXO1, NC_001496; pXO2 NC_002146). The statistical significance of linkage disequilibrium between site pairs was performed by using the Fisher's Exact Test at *P *< 10^-3^[69].

### Estimating levels of genetic variation

To account for missing data, θ is estimated by [Σ_n_(S_n_/a_n_)]/L, where S_n _is the number of observed segregating sites at positions with exactly n alleles sequenced (n is a maximum of 56, fewer with missing data), a_n _= Σ_i = 1..*n*-1 _1/i, and L is the total length of the sequence examined. Var{θ} is estimated by [Σ_n _(L_n_θ/a_n _+ (L_n_)^2^b_n_θ^2^/(a_n_)^2^]/L^2^, where L_n _is the number of sites with data from exactly n alleles, and b_n _= Σ_i = 1..*n*-1 _1/i^2^. With missing data π is estimated by [Σ_i _2p_i_q_i_n_i_/(n_i _- 1)]/L, where the sum is taken over all sites i, p_i _and q_i _are the allele frequencies at site i, and n_i _is the number of alleles sequenced at site i.

To determine if the estimates of theta between SNP types (silent, replacement, intergenic) are significantly different, we used the number of samples sequenced, the number of segregating sites, and the length of the region to find a maximum-likelihood estimate of theta per site for each SNP type using equations 11 and 12 in Hudson [70]. We compared all possible SNP types against each other (silent vs replacement, silent vs intergenic, replacement vs. intergenic). For a given pair of SNP types, we first determined the maximum-likelihood estimator of theta for each type individually. We then determined the maximum-likelihood estimator of theta, assuming both types had identical theta per site. We ask whether the model with different thetas for each type fits significantly better than the model with a single theta through a likelihood ratio test. Reported significances are the *p*-values from the likelihood ratio test.

### Site frequency spectrum

Comparing the observed site frequency spectrum with that expected under the neutral theory is a powerful approach to detect unusual patterns of genetic diversity. We employed two different approaches for this analysis. First, we calculated the expected number of sites with minor allele frequency i as Σ_n_θL_n _[1/i + 1/(n - i)] and from this determine the expected percent of sites under the neutral expectation. This is directly compared with the observed percent of SNPs in Figure 2. Confidence intervals for the sample proportion of each SNP minor allele frequency classes as



where N is the number of SNPs observed for each class,  is their observed frequency, and .

As a second method, we employed Tajima's D statistic [50], estimated as (π - θ)/Var(π - θ). Under the neutral model, π and θ have the same expectation, hence Tajima's D is expected to be 0. Since π is a function of site heterozygosities and θ is a function of the total number of segregating sites, Tajima's D is negative (positive) with an excess (deficit) of rare sites. We use our estimated values of π [51] and θ [45], multiplied by the total genome *B. anthracis *genome length (5,505,178), to determine the expected number of SNPs that we would expect to observe among two *B. anthracis *strains sampled in same random fashion as isolates in this study were chosen. Using Equations 6-9 in [51], we calculated the variance of  and θ estimators. The one standard deviation (SD) that we report is the square root of this variance.

### Phylogenetic tree inference

The 37 variable positions identified in this study were concatenated together to create artificial sequence types. A DNA distance matrix was created using DNADIST, plotted as a UPGMA tree using NEIGHBOR and the tree plotted using DRAWGRAM [57].

## Additional data files

The following additional data are available with the online version of this article. Additional data file 1 lists *B. anthracis *strains from the Biological Defense Research Directorate (BDRD) strain collection resequenced in this study. Additional data file 2 lists the BDRD-01 RA fragment names, the GenBank reference sequence from which they are derived, the length of the unique genomic sequences submitted to RA design, the length of the unique genomic sequences capable of being queried, and the LPCR primer pairs used to amplify the RA fragments. Additional data file 3 lists the *B. anthracis *SNPs identified in this study. The data include the BDRD SNP ID, the GenBank reference sequence and RA fragment containing the SNP, the SNP position relative to the GenBank reference sequence and the RA sequence, the SNP frequency, and the listing of the base calls in all strains at sites harboring SNPs. Additional data file 4 lists the 31 *B. anthracis *genes partially or wholly resequenced in this study. The observed number SNPs by SNP type (silent vs replacement) for each gene are provided. Finally, Additional data file 5 shows the genomic sequences submitted to RA design for BDRD-01.

## Supplementary Material

Additional data file 1 *B. anthracis *strains from the Biological Defense Research Directorate (BDRD) strain collection resequenced in this study Click here for additional data file

Additional data file 2 The BDRD-01 RA fragment names, the GenBank reference sequence from which they are derived, the length of the unique genomic sequences submitted to RA design, the length of the unique genomic sequences capable of being queried, and the LPCR primer pairs used to amplify the RA fragments Click here for additional data file

Additional data file 3 The *B. anthracis *SNPs identified in this study. The data include the BDRD SNP ID, the GenBank reference sequence and RA fragment containing the SNP, the SNP position relative to the GenBank reference sequence and the RA sequence, the SNP frequency, and the listing of the base calls in all strains at sites harboring SNPs Click here for additional data file

Additional data file 4 The 31 *B. anthracis *genes partially or wholly resequenced in this study Click here for additional data file

Additional data file 5 The genomic sequences submitted to RA design for BDRD-01 Click here for additional data file

## Figures and Tables

**Table 1:** Assessing microarray resequencing data quality

**Replication experiment**	
Total number of bases called in replicate 1	1,383,229
Total number of bases called in replicate 2	1,373,905
Total number of bases called in both replicates	1,349,177
Total number of bases called differently	1
Replication experiment discrepancy rate	7.4E-07
	
**Accuracy estimation experiment**	
Total number of bases called identically	398,452
Total number of bases called differently	15
Accuracy experiment discrepancy rate	3.8E-05

**Table 2:** Observed genetic variation in *B. anthracis*

	Observed number of SNPs	Total amount resequenced (bp)	Nucleotide diversity (× 10^4^) ± 2 SEs	Tajima's D
**Total**	37	1,544,913	2.9 ± 1.3	-0.93
				
**Genomic location**				
Chromosome	18	874,564	2.5 ± 1.4	-0.95
pXO1	9	325,397	3.3 ± 2.4	-0.54
pXO2	10	344,952	3.5 ± 2.5	-0.73
				
**SNP class**				
Silent	15	243,481	7.5 ± 4.3	-0.55
Replacement	9	898,837	1.2 ± 0.80	-0.64
Intergenic	13	402,595	3.8 ± 2.3	-1.09

**Table 3:** Observed patterns of polymorphism/divergence between *B. anthracis *(Ames) and *B. cereus *(ATCC 14579, ATCC 10987)

	Silent sites	Replacement sites
Polymorphic sites within *B. cereus *strains	660	136
Divergent sites between *B. anthracis *and *B. cereus*	646	125
Polymorphic sites within *B. anthracis *strains	11	3
