# Supplementary material for: Microarray-based resequencing of multiple Bacillus anthracis isolates
Source: Genome Biol. 2004 Dec 17;6(1):R10. doi: 10.1186/gb-2004-6-1-r10 (PMC549062; doi:10.1186/gb-2004-6-1-r10)
Supplement: Additional data file 1 — B. anthracis strains from the Biological Defense Research Directorate (BDRD) strain collection resequenced in this study [file gb-2004-6-1-r10-s1.pdf]

Additional Data 1

***B. anthracis* Strains Resequenced**

Strain designation	Brief Description	MLVA Genotype	MLVA Cluster
ASC004	Strain M36; used in vaccine research, U. K.	Strains not genotyped by MLVA	
ASC006	Vollum 3b type strain. U.K.		
ASC010	NCTC 2620. China.		
ASC014	ATCC 241.		
ASC015	ATCC 938.		
ASC016	ATCC 937.		
ASC025	U.K. bovine case presumed to be caused by contaminated material from Senegal.		
ASC027	U.K. bovine case presumed to be caused by contaminated material from Senegal.		
ASC031	U.K. bovine case presumed to be caused by contaminated material from Senegal.		
ASC032	Penicillin-resistant fatal human case. U. K.		
ASC038	Fatal human case. U.K.		
ASC050	Zimbabwe (Human cutaneous isolate).		
ASC054	Zimbabwe (Human cutaneous isolate). Phage resistant.		
ASC061	Zebra. Etosha National Park. Namibia.		
ASC065	Cow. Brazil. Uncharacteristic colony form.		
ASC069	Human isolate. New Hampshire, U.S.A.		
ASC070	Penicillin resistant.		
ASC073	Zebra. Etosha N. P. Namibia.		
ASC074	Vulture feces, Etosha NP, Namibia.		
ASC120	Australia.		
ASC131	Elephant skull. Zambia.		
ASC152	Giraffe bone. Namibia.		
ASC158	Zebra. Etosha NP Namibia,		
ASC159	Ames. Guinea pig re-isolate from vaccine challenge studies. U.K.		
ASC161	Ames. Guinea pig re-isolate from vaccine challenge studies. U.K.		
ASC165	Ames. Guinea pig re-isolate from vaccine challenge studies. U.K.		
ASC206	Kruger N. P. South Africa.		
ASC254	Environmental isolate. U.K. Believed to be more than 100 years old.		
ASC285	Environmental isolate. U.K. Believed to be more than 100 years old.		

ASC330	Ames re-isolate. U.K.		
ASC386	Ames re-isolate with uncharacteristic colony morphology. U.K.		
ASC394	Ames re-isolate from guinea pig which died despite ciprofloxacin treatment. U.K.		
ASC398	Ames re-isolate from guinea pig which died despite doxycycline treatment. U.K.		
BDRD_01	Unknown A0089 strain		
A0034	Bovine. China.	57	A3b
A0039	Bovine. Australia.	55	A3a
A0149	Human cutaneous isolate. Turkey.	23	A1b
A0158	Bovine. Zambia.	30	A3a
A0174	Canada	3	A1a
A0188	Zebra. Etosha N.P. Namibia.	35	A3a
A0193	Bovine. U.S.A.	10	A1b
A0248	Human. U.S.A.	68	A3d
A0256	Human. Turkey.	41	A3a
A0264	Human. Turkey.	28	A1b
A0267	Bovine. U.S.A.	25	A1b
A0293	Sheep. Italy.	20	A1a
A0328	Pig. Germany.	38	A3a
A0376	Bovine. U.S.A.	51	A3a
A0379	Wool. Pakistan.	69	A4
A0419	South Korea (fatal human case).	34	A3a
A0442	Kudu, Kruger N.P. South Africa.	87	B2
A0462	Ames Guinea pig re-isolate from vaccine challenge studies (Porton Down U. K.).	62	A3b
A0463	Sheep. Pakistan.	29	A2
A0488	U.K. (Vollum).	77	A4
A0465	Bovine. France.	80	B1
A0489	Bovine. Argentina.	45	A3a
