# Supplementary material for: Microarray-based resequencing of multiple Bacillus anthracis isolates
Source: Genome Biol. 2004 Dec 17;6(1):R10. doi: 10.1186/gb-2004-6-1-r10 (PMC549062; doi:10.1186/gb-2004-6-1-r10)
Supplement: Additional data file 2 — The BDRD-01 RA fragment names, the GenBank reference sequence from which they are derived, the length of the unique genomic sequences submitted to RA design, the length of the unique genomic sequences capable of being queried, and the LPCR primer pairs used to amplify the RA fragments [file gb-2004-6-1-r10-s2.pdf]

Additional Data 2

BDRD-01 Resequencing Array (RA) Sequence and Primer Information

BDRD-01 RA Fragment Name	GenBank Reference Sequence	Genomic Sequence Submitted to RA Design	Genomic Sequence Capable of Being Queried	LPCR Primer Pairs Used to Amplify Fragment
nmrc_001	NC_001496	953	929	ant8 / ant9
nmrc_002	NC_001496	607	583	ant10 / ant11
nmrc_003	NC_001496	1,520	1,496	ant10 / ant11
nmrc_004	NC_001496	1,280	1,256	ant10 / ant11
nmrc_005	NC_001496	1,831	1,807	ant10 / ant11
nmrc_006	NC_002146	771	747	ant14 / ant15
nmrc_007	NC_002146	5,954	5,930	ant14 / ant15
nmrc_008	NC_003997	3,053	3,029	ant20 / ant21
nmrc_009	NC_003997	2,106	2,082	ant20 / ant21
nmrc_010	NC_003997	6,077	6,053	ant26 / ant27
nmrc_011	NC_003997	2,709	2,685	ant32 / ant33
nmrc_012	NC_003997	2,639	2,615	ant32 / ant33
		29,500	29,212	
