# Supplementary material for: Microarray-based resequencing of multiple Bacillus anthracis isolates
Source: Genome Biol. 2004 Dec 17;6(1):R10. doi: 10.1186/gb-2004-6-1-r10 (PMC549062; doi:10.1186/gb-2004-6-1-r10)
Supplement: Additional data file 3 — The B. anthracis SNPs identified in this study. The data include the BDRD SNP ID, the GenBank reference sequence and RA fragment containing the SNP, the SNP position relative to the GenBank reference sequence and the RA sequence, the SNP frequency, and the listing of the base calls in all strains at sites harboring SNPs [file gb-2004-6-1-r10-s3.pdf]

Additional Data 3

***B. anthracis* Single Nucleotide Polymorphisms (SNPs) Identified**

SNP ID	GenBank Reference Sequence	RA Fragment Identification	SNP Position · Relative to RA Sequence	SNP Position Relative to Genbank Reference Sequence	SNP Frequency	ASC004	ASC006	ASC010	ASC014	ASC015	ASC016	ASC025	ASC027	ASC031	ASC032	ASC038	ASC050	ASC054	ASC061	ASC065	ASC069	ASC070	ASC073	ASC074	ASC120	ASC131	ASC152	ASC158
1	NC_001496	nmrc_003	871	129567	0.156	G	A	N	A	A	A	A	G	A	A	A	G	G	A	A	A	A	A	A	N	N	N	A
2	NC_001496	nmrc_004	120	132396	0.125	A	G	G	G	G	G	G	A	G	G	G	A	A	G	G	G	G	G	G	G	G	G	G
3	NC_001496	nmrc_004	122	132398	0.018	A	A	A	A	A	A	A	A	A	A	A	A	A	A	A	A	A	A	A	A	A	A	A
4	NC_001496	nmrc_004	124	132400	0.018	A	A	A	A	A	A	A	A	A	A	A	A	A	A	A	A	A	A	A	A	G	A	A
5	NC_001496	nmrc_004	160	132436	0.036	T	C	T	T	T	T	T	T	T	T	T	T	T	T	T	T	T	T	T	T	T	T	T
6	NC_001496	nmrc_004	356	132632	0.036	G	A	G	G	G	G	G	G	G	G	G	G	G	G	G	G	G	G	G	G	G	G	G
7	NC_001496	nmrc_005	38	133691	0.036	G	G	G	G	G	G	G	G	G	G	G	G	G	G	G	G	G	G	G	G	G	G	G
8	NC_001496	nmrc_005	110	133763	0.071	G	A	G	G	G	G	G	G	G	G	G	G	G	G	G	A	G	G	G	G	G	G	G
9	NC_001496	nmrc_005	1608	135261	0.491	A	A	G	G	G	N	G	A	G	G	G	A	A	G	A	A	G	G	G	G	A	G	G
10	NC_002146	nmrc_006	21	49156	0.115	C	T	T	T	T	T	T	C	T	T	T	C	C	T	N	N	T	T	T	T	T	T	T
11	NC_002146	nmrc_006	351	49486	0.094	T	C	T	T	T	T	T	T	T	T	T	T	T	T	N	C	T	T	T	T	T	T	T
12	NC_002146	nmrc_007	339	51207	0.109	T	C	C	C	C	C	C	T	C	C	C	T	T	C	C	C	C	C	C	C	C	C	C
13	NC_002146	nmrc_007	783	51651	0.055	T	T	T	T	T	T	T	T	T	T	T	T	T	T	T	T	T	T	T	T	T	T	T
14	NC_002146	nmrc_007	1303	52171	0.020	T	T	T	T	T	T	T	T	T	T	T	T	T	T	T	T	T	T	T	T	T	T	T
15	NC_002146	nmrc_007	2137	53005	0.109	G	A	A	A	A	A	A	G	A	A	A	G	G	A	A	A	A	A	A	A	A	A	A
16	NC_002146	nmrc_007	2661	53529	0.020	C	C	C	C	C	N	C	C	C	C	C	N	C	C	C	C	C	C	C	C	C	C	C
17	NC_002146	nmrc_007	2833	53701	0.027	A	N	A	A	A	N	A	A	A	A	A	A	A	N	N	N	A	A	N	N	A	N	N
18	NC_002146	nmrc_007	3223	54091	0.019	A	A	A	A	A	A	A	A	A	A	A	A	A	A	N	A	A	A	A	A	A	A	A
19	NC_002146	nmrc_007	3713	54581	0.115	C	T	N	T	T	T	T	C	T	T	T	C	C	T	N	N	T	T	T	T	T	T	T
20	NC_003997	nmrc_008	601	4099657	0.019	N	C	C	C	C	C	C	C	C	N	C	C	C	C	C	C	C	C	C	C	G	C	C
21	NC_003997	nmrc_008	792	4099848	0.113	N	T	T	T	T	T	T	G	T	N	T	G	G	T	T	T	T	T	T	T	T	T	N
22	NC_003997	nmrc_008	1248	4100304	0.025	N	N	T	T	T	T	T	T	T	N	T	T	T	T	N	N	T	T	T	T	N	T	T
23	NC_003997	nmrc_008	2201	4101257	0.019	N	A	A	A	A	A	A	A	A	N	A	A	A	A	A	A	A	A	A	A	A	A	A
24	NC_003997	nmrc_009	1263	4105216	0.113	N	T	T	T	T	T	T	C	T	N	T	C	C	T	N	T	T	T	T	T	T	T	T
25	NC_003997	nmrc_010	208	104420	0.023	T	N	N	T	T	T	T	T	T	T	T	T	T	T	N	N	T	T	T	T	T	T	T
26	NC_003997	nmrc_010	3748	107960	0.091	G	A	A	A	A	A	A	G	A	A	A	G	G	A	A	A	A	A	A	A	A	A	A
27	NC_003997	nmrc_010	3916	108128	0.054	C	C	C	C	C	C	C	C	C	C	C	C	C	C	C	C	C	C	C	C	C	C	C
28	NC_003997	nmrc_010	3967	108179	0.071	T	C	T	T	T	T	T	T	T	T	T	T	T	T	T	C	T	T	T	T	T	T	T
29	NC_003997	nmrc_010	4537	108749	0.054	C	C	C	C	C	C	C	C	C	C	C	C	C	C	C	C	C	C	C	C	C	C	C
30	NC_003997	nmrc_010	4772	108984	0.018	A	A	A	A	A	A	A	A	A	A	A	A	A	A	A	A	A	A	A	G	A	A	A
31	NC_003997	nmrc_010	5091	109303	0.018	G	G	G	G	G	G	G	G	G	G	A	G	G	G	G	G	G	G	G	G	G	G	G
32	NC_003997	nmrc_010	5939	110151	0.018	T	T	T	A	N	T	T	T	T	T	T	T	T	T	T	T	T	T	T	T	T	T	T
33	NC_003997	nmrc_011	62	511175	0.091	T	T	T	T	T	T	T	C	T	T	T	C	C	T	N	T	T	T	T	T	T	T	T
34	NC_003997	nmrc_011	649	511762	0.018	G	G	G	G	G	G	G	G	G	G	G	G	G	G	G	G	G	G	G	G	G	G	G
35	NC_003997	nmrc_011	1939	513052	0.018	T	C	C	C	C	C	C	C	C	C	C	C	C	C	C	C	C	C	C	C	C	C	C
36	NC_003997	nmrc_012	1251	515111	0.185	A	A	G	A	A	A	A	A	A	A	A	A	A	A	N	A	A	A	A	A	A	A	G
37	NC_003997	nmrc_012	1851	515711	0.304	A	A	A	A	A	A	G	A	G	G	G	A	A	G	A	A	G	G	G	G	A	G	A

ASC159	ASC161	ASC165	ASC206	ASC254	ASC285	ASC330	ASC386	ASC394	ASC398	A0034	A0039	A0089	A0149	A0158	A0174	A0188	A0193	A0248	A0256	A0264	A0267	A0293	A0328	A0376	A0379	A0419	A0442	A0462	A0463	A0488	A0465	A0489
A	N	A	G	N	N	A	A	A	A	A	A	A	A	A	A	A	N	A	A	A	N	A	A	A	A	N	G	A	A	G	A	N
G	G	G	A	G	G	G	G	G	G	G	G	G	G	G	G	G	G	G	G	G	G	G	G	G	G	G	A	G	G	A	G	G
A	A	A	A	A	A	A	A	A	A	A	A	A	A	A	A	A	A	A	A	A	A	A	A	A	A	G	A	A	A	A	A	A
A	A	A	A	A	A	A	A	A	A	A	A	A	A	A	A	A	A	A	A	A	A	A	A	A	A	A	A	A	A	A	A	A
T	T	T	T	T	T	T	T	T	T	T	N	T	T	T	T	T	T	T	T	T	T	T	T	T	T	T	T	T	T	T	C	T
G	G	G	G	G	G	G	G	G	G	G	G	G	G	G	G	G	G	G	G	G	G	G	G	G	G	G	G	G	G	G	A	G
G	G	G	T	G	G	G	G	G	G	G	G	G	G	G	G	G	G	G	G	G	G	G	G	G	G	G	T	G	G	G	G	G
G	G	G	G	G	G	G	G	G	G	G	G	G	A	G	G	G	G	G	G	G	G	G	G	G	G	G	G	G	G	G	A	G
G	G	G	A	A	N	G	A	G	G	G	A	A	A	G	G	A	A	N	A	G	G	A	A	G	A	A	A	G	A	A	A	A
T	T	T	C	T	T	T	T	T	T	T	T	N	T	T	T	T	T	T	T	T	T	T	T	T	T	T	C	T	T	T	T	N
T	T	T	T	T	T	T	C	T	T	T	T	N	C	T	T	T	T	T	T	T	T	T	T	T	T	T	T	T	T	T	C	N
C	C	C	T	C	C	C	C	C	C	C	C	N	C	C	C	C	C	C	C	C	C	C	C	C	C	C	T	C	C	C	C	C
T	T	T	T	T	T	T	T	T	T	T	C	N	T	T	T	T	T	T	T	T	T	C	T	T	T	T	T	T	C	T	T	T
T	T	T	T	T	T	T	T	T	T	T	T	N	T	T	T	T	C	N	T	T	T	N	T	T	N	T	T	N	T	T	T	T
A	A	A	G	A	A	A	A	A	A	A	A	N	A	A	A	A	A	A	A	A	A	A	A	A	A	A	G	A	A	A	A	A
C	C	C	C	C	C	C	C	C	C	C	C	N	C	C	C	C	C	C	C	N	C	C	C	C	C	T	N	C	C	C	C	C
A	N	A	A	A	N	N	A	A	A	A	A	N	N	A	A	A	A	A	A	A	A	N	A	A	A	T	N	N	N	A	N	A
A	A	A	A	A	A	A	A	A	A	A	A	N	A	A	A	A	A	A	A	A	A	A	A	A	A	A	A	A	A	G	A	A
T	T	T	C	T	T	T	T	T	T	T	T	N	T	T	T	T	T	T	T	T	T	T	T	T	T	T	C	T	T	T	T	T
C	C	C	C	C	C	C	C	C	C	C	C	C	C	C	C	C	C	C	C	C	C	C	C	C	C	C	C	C	C	C	C	C
T	T	T	G	T	T	T	T	T	T	T	T	T	T	T	T	T	T	T	T	T	T	T	T	T	T	T	G	T	T	G	T	T
T	T	T	T	T	N	T	T	T	T	T	T	T	T	N	T	N	T	N	N	T	T	T	A	T	N	N	T	N	T	T	N	N
A	A	A	A	A	A	A	A	A	A	A	A	A	A	A	A	A	A	A	A	G	A	A	A	A	A	A	A	A	A	A	A	A
T	T	T	C	T	T	T	T	T	T	T	T	T	T	T	T	T	T	T	T	T	T	T	T	T	T	T	C	T	T	C	T	T
T	T	T	T	T	T	T	T	T	T	T	T	T	T	T	N	N	T	N	T	T	T	N	N	A	N	T	T	N	N	T	N	T
A	A	A	G	A	A	A	A	A	A	A	A	A	A	A	A	A	A	A	A	A	A	A	A	A	A	A	N	A	A	A	A	A
C	C	C	C	C	C	C	C	C	C	C	C	A	C	C	C	C	C	C	C	C	C	C	C	C	A	C	C	C	C	C	C	A
T	T	T	T	T	T	T	T	T	T	T	T	T	C	T	T	T	T	T	T	T	T	T	T	T	T	T	T	T	T	T	C	T
C	C	C	C	C	C	C	C	C	C	C	A	C	C	C	C	C	C	C	C	C	C	A	C	C	C	C	C	C	A	C	C	C
A	A	A	A	A	A	A	A	A	A	A	A	A	A	A	A	A	A	A	A	A	A	A	A	A	A	A	A	A	A	A	A	A
G	G	G	G	G	G	G	G	G	G	G	G	G	G	G	G	G	G	G	G	G	G	G	G	G	G	G	G	G	G	G	G	G
T	T	T	T	T	T	T	T	T	T	T	T	T	T	T	T	T	T	T	T	T	T	T	T	T	T	T	T	T	T	T	T	T
T	T	T	C	T	T	T	T	T	T	T	T	T	T	T	T	T	T	T	T	T	T	T	T	T	T	T	C	T	T	T	T	T
G	G	G	G	G	G	G	G	G	G	G	G	G	G	G	G	G	G	G	G	G	G	G	G	G	G	A	G	G	G	G	G	G
C	C	C	C	C	C	C	C	C	C	C	C	C	C	C	C	C	C	C	C	C	C	C	C	C	C	C	C	C	C	C	C	C
G	G	G	A	A	A	G	N	A	G	A	A	A	A	G	G	A	A	A	A	A	A	A	A	A	A	A	A	G	A	A	A	A
A	A	A	A	A	G	A	A	G	A	G	A	A	A	A	A	A	A	G	A	G	G	A	A	G	A	A	A	A	A	A	A	A
