# Supplementary material for: Microarray-based resequencing of multiple Bacillus anthracis isolates
Source: Genome Biol. 2004 Dec 17;6(1):R10. doi: 10.1186/gb-2004-6-1-r10 (PMC549062; doi:10.1186/gb-2004-6-1-r10)
Supplement: Additional data file 4 — The 31 B. anthracis genes partially or wholly resequenced in this study [file gb-2004-6-1-r10-s4.pdf]

Additional Data 4

Gene Location, Length of, and SNPs Discovered in *B. anthracis* Resequenced Genes

GenBank Reference Sequence	Gene Product ID	Additional Gene Information	Total Gene Length	Exon Bases Resequenced	SNPs at Silent Sites	SNPs at Replacement Sites
NC_001496	pXO1-106	-	381	125	0	0
NC_001496	pXO1-107	Anthrax toxin lethal factor precursor; lef	2430	1993	0	1
NC_001496	pXO1-110	Anthrax toxin moiety, protective antigen, pagA	2295	2174	1	2
NC_002146	pXO2-53	Similar to Bacillus anthracis pXO2 postive trans-activator of capsule synthesis AcpA encoded by GenBank Accession Number U02535	1449	476	0	1
NC_002146	pXO2-54	-	144	144	0	0
NC_002146	pXO2-55-dep	Similar to B. anthracis pXO2 dep protein encoded by GenBank Accession Number D14037	1401	1401	3	1
NC_002146	pXO2-56-CapA	Similar to B. anthracis pXO2 CapA encoded by GenBank Accession Number M24150	1236	1236	0	1
NC_002146	pXO2-57-CapC	Similar to B. anthracis pXO2 CapC encoded by GenBank Accession Number M24150	450	450	0	0
NC_003997	BA4505	Cation ABC transporter, ATP-binding protein, putative	771	407	0	0
NC_003997	BA4506	Membrate protein, putative	879	879	2	0
NC_003997	BA4507	Conserved hypothetical protein	255	255	0	0
NC_003997	BA4508	Endonuclease IV	897	897	1	0
NC_003997	BA4510	vrrA protein	769	440	0	0
NC_003997	BA4511	penicillin tolerance protein LytB	951	951	1	0
NC_003997	BA0096	Transcription antitermination protein NusG	534	534	1	0
NC_003997	BA0097	Ribosomal protein L11	426	426	0	0
NC_003997	BA0098	Ribosomal protein L1	693	693	0	0
NC_003997	BA0099	Ribosomal Protein L10	501	501	0	0
NC_003997	BA0100	Ribosomal Protein L7/L12	360	360	0	0
NC_003997	BA0101	ybxB protein	600	600	1	0
NC_003997	BA0102	DNA-directed RNA polymerase, beta subunit	3534	1886	2	2
NC_003997	BA0515	Cell Division inhibitor-like protein	906	676	1	1
NC_003997	BA0516	recX domain protein	813	813	0	0
NC_003997	BA0517	Conserved hypothetical protein	312	312	0	0
NC_003997	BA0518	Hypothetical protein	153	153	0	0
NC_003997	BA0519	Conserved hypothetical protein	159	159	0	0
NC_003997	BA0520	Conserved Hypothetical Protein	267	248	0	0
NC_003997	BA0521	yfhP protein	981	962	0	0
NC_003997	BA0522	A/G-specific adenine glycosylase	1098	1098	2	0
NC_003997	BA0523	yfhS protein	288	288	0	0
NC_003997	BA0524	Small acid-soluble spore protein, gamma-type	288	22	0	0
