# Supplementary material for: Microarray-based resequencing of multiple Bacillus anthracis isolates
Source: Genome Biol. 2004 Dec 17;6(1):R10. doi: 10.1186/gb-2004-6-1-r10 (PMC549062; doi:10.1186/gb-2004-6-1-r10)
Supplement: Additional data file 5 — The genomic sequences submitted to RA design for BDRD-01 [file gb-2004-6-1-r10-s5.pdf]

Additional Data 5

Genomic Sequences Submitted to RA Design for BDRD-01

>nmrc_001
ATTAAAAAATTATATGCTATGTCAATTATATTCATAGCGCGTTTTTACCGTTATAAAAGATAAAAACTCGAAAACAGAA
AAATAATATACCTTTTATTCTATACAGCAACCTAAATATTATAATCAACTTTTCCATAGAAATTAATCCTTTTGTTATAC
ATCTTTATTCCCTTAACATTGTCAAATTTCAGTTATTCATTCTGGATAGTCAATAAATAGATTACGGTTATGTTAGTATT
TTTTTAAAATAATAGTATTAAATAGTGGAATGCAAATGATAAATGGGCTTTAAACAAAACTAATGAAATAATCTACAAAT
GGAATTTCTCCAGTTTTAGATTAAACCATACCAAAAAAATCACACTGTCAAGAAAAATGATAGAATCCCTACACTAATTA
ACATAACCAAATTGGTAGTTATAGGTAGAAACTTATTTATTTCTATAATACCATGCAAAAAACAAACTAAATATTCTGTT
CCATACTATTTTAGTAAATTATTTAGCAAGTAAATTTTGGTGTATAAACAAAGTTTATCTTAATATAAAAAATTACTTTA
CTTTTATACAGATTAAAATGAAAAATTTTTTATGACAAGAAATATTGCCTTTAATTTATGAGGAAATAAGTAAAATTTTC
TACATACTTTATTTTATTGTTGAAATGTTCACTTATAAAAAAGGAGAGATTAAATATGAATATAAAAAAAGAATTTATAA
AAGTAATTAGTATGTCATGTTTAGTAACAGCAATTACTTTGAGTGGTCCCGTCTTTATCCCCCTTGTACAGGGGGCGGGC
GGTCATGGTGATGTAGGTATGCACGTAAAAGAGAAAGAGAAAAATAAAGATGAGAATAAGAGAAAAGATGAAGAACGAAA
TAAAACACAGGAAGAGCATTTAAAGGAAATCATGAAACACATTGTAAAAATAGAAGTAAAAGGGGAGGAAGCT
>nmrc_002
ATATATATTGTGGATGGTGATATTACAAAACATATATCTTTAGAAGCATTATCTGAAGATAAGAAAAAAATAAAAGACAT
TTATGGGAAAGATGCTTTATTACATGAACATTATGTATATGCAAAAGAAGGATATGAACCCGTACTTGTAATCCAATCTT
CGGAAGATTATGTAGAAAATACTGAAAAGGCACTGAACGTTTATTATGAAATAGGTAAGATATTATCAAGGGATATTTTA
AGTAAAATTAATCAACCATATCAGAAATTTTTAGATGTATTAAATACCATTAAAAATGCATCTGATTCAGATGGACAAGA
TCTTTTATTTACTAATCAGCTTAAGGAACATCCCACAGACTTTTCTGTAGAATTCTTGGAACAAAATAGCAATGAGGTAC
AAGAAGTATTTGCGAAAGCTTTTGCATATTATATCGAGCCACAGCATCGTGATGTTTTACAGCTTTATGCACCGGAAGCT
TTTAATTACATGGATAAATTTAACGAACAAGAAATAAATCTATCCTTGGAAGAACTTAAAGATCAACGGATGCTGTCAAG
ATATGAAAAATGGGAAAAGATAAAACAGCACTATCAACACTGGAGCG
>nmrc_003
TATGATATTAATCAAAGGTTGCAAGATACAGGAGGGTTAATTGATAGTCCGTCAATTAATCTTGATGTAAGAAAGCAGTA
TAAAAGGGATATTCAAAATATTGATGCTTTATTACATCAATCCATTGGAAGTACCTTGTACAATAAAATTTATTTGTATG
AAAATATGAATATCAATAACCTTACAGCAACCCTAGGTGCGGATTTAGTTGATTCCACTGATAATACTAAAATTAATAGA
GGTATTTTCAATGAATTCAAAAAAAATTTCAAATATAGTATTTCTAGTAACTATATGATTGTTGATATAAATGAAAGGCC
TGCATTAGATAATGAGCGTTTGAAATGGAGAATCCAATTATCACCAGATACTCGAGCAGGATATTTAGAAAATGGAAAGC
TTATATTACAAAGAAACATCGGTCTGGAAATAAAGGATGTACAAATAATTAAGCAATCCGAAAAAGAATATATAAGGATT
GATGCGAAAGTAGTGCCAAAGAGTAAAATAGATACAAAAATTCAAGAAGCACAGTTAAATATAAATCAGGAATGGAATAA
AGCATTAGGGTTACCAAAATATACAAAGCTTATTACATTCAACGTGCATAATAGATATGCATCCAATATTGTAGAAAGTG
CTTATTTAATATTGAATGAATGGAAAAATAATATTCAAAGTGATCTTATAAAAAAGGTAACAAATTACTTAGTTGATGGT
AATGGAAGATTTGTTTTTACCGATATTACTCTCCCTAATATAGCTGAACAATATACACATCAAGATGAGATATATGAGCA
AGTTCATTCAAAAGGGTTATATGTTCCAGAATCCCGTTCTATATTACTCCATGGACCTTCAAAAGGTGTAGAATTAAGGA
ATGATAGTGAGGGTTTTATACACGAATTTGGACATGCTGTGGATGATTATGCTGGATATCTATTAGATAAGAACCAATCT
GATTTAGTTACAAATTCTAAAAAATTCATTGATATTTTTAAGGAAGAAGGGAGTAATTTAACTTCGTATGGGAGAACAAA
TGAAGCGGAATTTTTTGCAGAAGCCTTTAGGTTAATGCATTCTACGGACCATGCTGAACGTTTAAAAGTTCAAAAAAATG
CTCCGAAAACTTTCCAATTTATTAACGATCAGATTAAGTTCATTATTAACTCATAAGTAATGTATTAAAAATTTTCAAAT
GGATTTAATAATAATAATAATAATAATAATAACGGGACCAGCCATTATGAAGCAACTAATTCTAGACTTGATAGTAATTC
TTGGGAAGCACCAGATAGTGTAAAAGGTGGCATTGCCAGAATGATATTTTATGTGTTCGTTAGATATGAAGGCAAAAACA
ATGATCCTGACCTAGAACTTAATGATAATGTTATTAATAATTTAATGCCTTTTATAGGAATATTAGTAAAAGTGCCGAAA
AGATCCTGTTGCAAAGCTTTTAAAGAACATATTATTCTATCAAGTGGCTGTATATTTTGTGTAATTTTCAATAAATTTTG
>nmrc_004
CCTTTTAATAATATGACATTATTATGTTCGTACATTGTGATGTTTTGTATAATAAACTGATTATATAATTCTAATGTATA
ATATTAATATGATTTTAATATTAAGTTTTTAAGAACTTTCGCACACTATTTTTAATATCTAATTTAGGATTTTTATAGTA
TTTTTAAATTAAATTATAAAGAAATAAAACAAGAAATGTATAGATTCTTTAACCTCATATTTTCTATTAATTGATTTAGA
GTATAGTCCCTATATCGTGATAAATTATATAAACTATATTCCTAAATTACCACTTTTATCTTTTCCAACAGAAGAATATT
TTTGGCAGTTGCTTTCTCTTTCAATATATTTGCCACGCTCTTGTGGTCCTTATCAATTTCAATCATACTATCTATTTACC
GTATTAACTTTTGCTCATTAATGTAGTGTAATTAGTTCATTGGCGCTCGTTTCGTCTAATCTACTAACTACCTATAATAA
TTAAAGTCTTTTCGCAATATAGATTTCTGCTATGCTGTTAGTAAGACACCCTTACCTTAATACATAGAACCGTTTCTGCA
CCTGAACAGCACACCTTTACTACATTCCTTAAGGCATGAACCTTTATTTGAGCATCAATATATTATAGATATCTTATTG
AATGTGCAATTGTCCTCTAATTAAATTTTTCTGTGCTTTCTAAACTCCAGATACTTCGCACAATTGTATATAGAATCATT
GGATGTTTCATTTTCTCTAAGTTTTTATGGATCCTACAAACAATCTCAAAGGATTATGATGATTTAGATTACTGTTTAAA
ACATACTCTCCTTGTATTTCTTACAAAAAGAGTATGTTTTAATTTTACTGTTTTTAGATAATTTAAAAATCACCTAGAA
TTACCTTATCCTATCTCATAGCCTTTTTTAGAAAAGATTAAAATTTCTTGATCCCGTTGGTACTAGTATCCCCATTCTC
ACTAGGATTAATAATAGTGTTTTCTTTAGTAACAGCATATACATTTACCTTATAATTGGGATTACTTATATATAACGGTA

```
ATTTATCATTATATTTTTTAAAATCTATAAATGTTTTTCCATCTTGCCGTAAACTAGAAATATTCAACATATCATATCTG
TCATTTATAACTTCTTTAAGCCCTTCAGTATCTTCAATTTCTACAATATAACCTGATAATATTTTTCTTATATCCTTATC
AATATTTAACAATAATCCCTCTGTTGACGAATTAATTACTTCTCTATGAGCCTCCTTAACTACTGACTCATCCGCCCCAA
>nmrc_005
AGTTGCGTTTAATTCCGCTAACTGATTCTTGATATTTTGAGATGTTTGTTGATCGAAATTAAAATCAAATTCGGTTATGT
CTTTCCCTTGATATTGTAAGTTTCCATTCGGTTCGTTAAATCCAAATGCTATTTTAAGGGCTTCTTTTAATGTCATATCC
GGTTTAGTCGTTTCTAATGGATCACTAGGATTAACCGCCGCTATCCGCCTTTCTACCAGATTTAAATCTTTTCCATTAAA
AATGATACGTGCAGTTGTTTCTTGAATTTGCGGTAACACTTCACTCCAGTTCGAGCCTGTATCCACCCTCACTCTTCCAT
TTTCAAAATTGTATGTTGCTATATTCCCATATACTTGATCCGTATCTAATCTTAATTGTTTCGTTTTTTCTAACTCAAGA
AATTGATTGTAATTCATTGTAATTGGAGTAGAACTGAAATCGTCTTGTGCATTTAATGCGATTGGCGCCAAGTTTTTAGA
AGGATAATAATTATTAGGTGCAAGTATTTGACTTAATTGGTTTTCCTTAGCTTTAATTGTCGCGAGTGTTTGATTTTTTC
CTAACACTAACGAAGTCGTTGGTAACACGTTGTAGATTGGAGCCGTCCCAGTATTTACATATCTAATATTGGCATTTAAT
CTTGCTGTATCAGCGGTATTTAAACCCATTGTTTCAGCCCAAGTTCTTTCCCCTGCTAGAGATAGTGAATGATCAATTGC
GACCGTACTTGAATTCGAATTACTAAATCCTGCAGATACACTCCCACCAATATCAAAGAACGACGCATGCACTTCTGCAT
TTCCATGTACTTCACTAGTATGTGTCCTACTTGTAGAAGTATTTTTACTTATTGTTCTCGTTTGACTATCAGTATTCTGT
GTGGATTGATCCTCATTTTTTGAGAGAATAATATTCTCCATATCTACATGTACAATCGGATAAGCTGCCACAAGGGGGTG
TCTTGCCTCTGGTGATACATTCTTATCAATCCGTCCTGTAACCTTTTCGAAATCACTGTACGGATCAGAAGCCGTGCTCC
ATTTTTCAGGAGATGATTTATATTTGGTTAATCCTTTCTTTTCATGAATATTAGAAATCCATGGTGAAAGAAAAGTTCTT
TTATTTTTGACATCAACCGTATATCCTTCTACCTCTAATGAATCAGGGATTCCATCATTGTCACGGTCTGGAACCGTAGG
TCCAGCACTTGTACTTCGCTTTTTTCTTGAGTTCGAAGATTTTTGTTTTAATTCTGGCAATTGTAAGTTATCACTAGAAA
TCACTTCTTTTTTATTTTGAGAATCGGTCCAGTACAACTTGAAATCCAATCCTTTTTCAGTAGGATTTTCTCGTTGATAT
TGAATTTTTATTTGATATAATCTTCCTTTTTCTAATCTGATTTTGTTAGAATTAGAAGCTTTATTAATCACTTCTTGGTC
ATCTACCCACATTGTTACATGATTATCAGCGGAAGTAGCAAATGTATATTCATCACTCTTCTTAACTTTGATAAATCCTG
ACCAAATAGCAGATTGAAAATATTGGTTTTCCGATGGAATATTTTCTAACTCAGAACTAGGAATAGATAAATCCCCTGTA
GTAGAAGAGGTAACCACCATGGGTGCTTGAAAATTCAAATCACTAAAATAGTATCCTAGTAACCCCTGGGAACTTGATTC
TGATTCATTTAATAACCGGTTCTCCTGTTTAACTTCTGCCTGAATCACCTCTAAATTACCTGTGCTTGAAACTAATATCG
TAGACAATGCCATTAATGGTATTAACACTTTTCGTTTTTTCATATACGTTCTCCTTTTTGTATAAAATTAA
>nmrc_006
AAGGACCTTGAGTAATCAAAGTAAACTAAAAAATCTCCTTTTTGTATACTGATATTTAGAAAAAAATTACTTAGCAGCTT
AGATATTTTTCCATTTTACAAAGAACAATCCATTTATTACTCACCATTTATACTTATAGTAAACCCTAGAAAAACTAAAA
ACATCAGGAAGTACCCCTATTCAGGAAACTTCTTATAAATATATATAGTTACTTCATTTTAAATAAAATCAAAAACAATA
ACAAAATAAAGCTTACAAAGTGGCTTTTTCTATATTATTGATACTAACCATCTTGTAAATCTAGATAATACTGTATATTG
GTGTAATCGCGTTCTGTAGGGATAGAATTAATACATATAATAGGGGTTGAACCTAAGTCAAGAGGTATTGTTGAAATAAT
AAAGTCAGCATTAACATCGTGATCACTCTTTTTAGCAAAAAAAACAGTTGAAATTTGTATTTTATCACCAAACCTTTTTG
CCAAAGTAGCAGCTAGATACTCTGCCCAACTTTCACTTTCTCCTGTGACAAGTAATACCTTTTTATAATTACTAGTATAA
CGAAGCCTACTTGCCTCTATATACATTGTAACTTTTGCAATTTCTTCAACAGATACATAGTCTGCTATCTCATTTTTTTT
GACAAATTCTGTGTATACTTCCTTTACTGCTGAGAAGGTTTCCGAATGTTCAGTTTGCATATACTGTATAGTTTGTTTTT
GCGGCCTTTCAAACATACATAGGTATTGTAAATGATATATCGTTCTTTTAA
>nmrc_007
TATCGATATCCCTTGCTTTTAAGAATGTAAATTACTTTTATACATATACAATTCTAGACAGTATAAGTAGAGGTAAAATT
AATTTCTTTACTCATTTTTGTTTGCAGAATGCAGTCAGAATCCCTGGTTTGTAATTATTAAGAACAAGGACATTTCCAAT
AAAAATATCCAATTTATATTCAAATTAATAATGTTACAATATTAATTAATATCACCTAATCATTAATATTTTTTATATCC
TTAGTTATATGCGTTTTTATTTTAGCAAGACATTTAACTGTTTTATAAAAAATTGCATTTCTGCCAAATAATATAATTAT
CATAAATAAAAATCAACATCCTTATTTGGTTATTATATAAATAGTCTCAGCCTATTACTTGCGATACATTTATTATTCTT
AGCATCTTAAATTCATAATGTTAAATCGCTTCCCCTCTAATCCTTTCCTAAAACTCTTAACGGCTTCGAGTTACCACCAT
TTCATATTTCAAATTAGCGATCTTTCTTAGTCAGTTTTTCTATTACTTCACGAGAAACTTTCTTCATTAATATTTCATTG
TAAGAACCAGTGTTAGGTGCGGTAACAGATATTATTGTTTGAACAATGATTTTTTACTTTTGCTTCATAATTTCCACTTC
ATATTACCACTATAATTTCTTTCAATTATGGTAAATAATAGATACCACAACTTAAATCTTAAATGCAATTCTCCTAAAAT
GTATTAGTTCTCAAACAAGGATTTTTTCTGCTTATGCAAGCTGTTTTGGAAAAGATTCAGCAGTGTTTCCAATTTTCATT
ATAATTTATAGAGTTTTTCAATAACCTAAAAAAACTTCCTTTGATTAAGAAGTTTTTTTGACTATTACAATAAAATTCAT
TTTTCAGCTAACATTCCCCATAAAAATATATATAAATTTTTTCTAATCACTCCTAAAGTCATTAATTTCAAATACATTTC
ATTCTACGATAATTTCCTCATTGGTGTTACTTTCCAGTAATTAAGACACCGTCAAATCCGTTCTACTGTACTATAATGAA
AATATTTTAACCACTGTTAAGATGCATATGCTAGCAATAGTTAGAGTTGGTTGTGATGTATCTTCACCAAGTAAAATCGG
CACAGTTGAATATTATGTCCAAATACAAAGAGAAGGTGAAATAAAAGGTAATGAACGTGTCTATACATTATTGACTTATG
ACAAAGAGGGAACAGAAAAGAAGGTCACATTTACTAGTCAAAAGCCTAATAATGAGAAGCTAAAAGAAAATGCATTTTTA
CGTCTTTATATTAAACAAGAGGATAAAGAGAAAGCAGACATACCTGATACAGAAGTTAAATCCTATGAAGAGATACAGAA
AGACGATTTACCAGCAAAAACAAAAGAACAATTAGGTGTTAAATAAAAGTATTGAGAATGACTTAAAAGGGTTCTCATAT
ATTAAGAGATACCCCTTTTAAGTTTATAAAATCAGAATATTATAAGATTTACTTTGTTTATTGGAATTATAAATTTTCCA
CAATAAAAAAGAATCATAAATTTATTAACTCGAATAATTTTTCGTTTAAATTGTATAATACTCCTAAAATAAATGACGGC
GTAACTTATTATCACAACAATTACCACTTCAATGGAAAAATCTGTAGAATGTAAGGAGGAACCCTCAAATAATTTTGAGT
```

AAATACTAAGCTTATTTGAATCCTGAGGATGGAATTCACAATTGGTATAATTAAAAAATATATCGAAAAATCTAAATCAC
TTTGTATACTTAGGGGTTAGCCTGTAGATAATCACTAATCTTCTTCTGCTCATCAGTCGTTAATGTTTCCGAACGTTTAA
AGGTCCCCATTGTTACAAGTAATAAACCTACAATTAAAATCGGCATAATCCATCCAAAAACTTTTTTAACCATTTCTCCA
TTACCTCCTATTTATTTGATTTCCAAGTTCCATTTCTTCTGCCATCTCCAGCGCCGGTGATAACATTATCTCTCTCGTCT
TTAATTAAGGCCTGTACCCCACCAAAAAAGGCAGGGGATACTTTCTTCTTTACGTTCAATCCTTTTCTAGATAACTCATT
TTTCACTTCTGAACTTAGCTGAATCTCTGTATACGCTGTATTTTTTTCAAAAGTAAAACGGTATTCATTGATAATGTCTT
GCAAGCTACCCTTACCATGCGTATATTTATCCAATATTGGGGTTAAAATTTGCGGAATACGGTTACCACCTGGTGACCCA
ATGCCGATCGTTTCCCCATCTTTCTTTAATACAGTGGGGGCCATAAACGTTCGTGAACGTTTACCAGGTTCATAATTATT
AAATCCCTCACTTCCAAAGTTCTGCAATTGATTATTTAAGAAGAACCCTGCTGTGTACTTTCCTGTTCCAAAGAAATTGC
TTAGTGTATTAGTTGAAGAGACAACCGTTCCATCTCTATCAATGATAACAAAATGCGTTGTGCTTTCATGCTCTGCTTCC
GAAAGCGCATCACCATTCTCATTCTTCATTGTTGATATATATTTGTCACTCACCATTTTATTTGGATCCATATTAACGTA
ATTTGGATCCCCTAGGTTTTTCTTTCTATCTTGATAGGCAATCCTTGAAATCTCTTCCATTTTAGACATATAAGTTGCCG
TATGATCTACATCTTTATATACTTCTTTCTTTTCAGCTAATTTCAACATTTGTAATAAAGTAACTCCTGAAAAAGGTGGT
GGAGCGGTATAAACATCATATCCCATGTAGTTACCTTTTACTGGTTTACGTACCTCTACTTTATATCCTTTTATATCTTC
TAACGATATTTTTGCAGTTTTACTGATTGCCCTAGCGACTCCTCCTTCATAAAAGCCTTTAGCCCCTTCTTTTTGAATCT
TCTTTAAGGTTCTCGCTAAATCTGTCTGGATAAGTGTTTCTCCAGTTTCAATAGGTTCACCATTCGGATAGAAGATACTT
AGCTTATCAGAATAAATACGTGGCTTCGCAAGGTCTAATCGCATTGTTAAGGAATCATCTACCTTGAACCCTTTTTCCGC
ATAATTAATGGCTGGTTGTAATAACTCACCCATCGGTAATGAACCATAATTATCATGAATATACTCCATTCCAGCCACAA
ATCCGGGTACTCCAATATGTGGCTTTTGGTTTCCTGTAAAGTACGGAGTTGTTTCACGATAATCAATAAAGGTTTCTTTA
TCTTTAGATATAATGAGCATTCCACCGCCCCCACCTATTCCTGAGGCATGCAGTTCTACAACGCCTAAAACATATGAGAC
CACAATCGCTGCATCTACTGCACTTCCACCGTTCTTTAATACTTTCATACCTTCCTCAACCGCAAGGGGGTGAGAGGCAC
TCACTCCATAAGTTCCTTTATCACCCATACTATCAATTTTTTGCTTAACACTGTCTTTTATTTTATTGAAAGAACAGGAT
ACCCCGATACCCCCCATTAAGCTGACTATCAAACAGAAAAGAATTATCTTCTTTCCCCATTTAAAGGAATTCAAGTTGTT
GTCTCCACTGATACTTGATTTTCTTTTTCTTGCTTATCTTGATGCTCTTGCTTTTCCCTCTTTTTCATTTTTTCAATAAC
ATGTTTATGATTTAATTTGATTTCCAATTTATCATCTTTTTTACTCCATAGAGCACCCTTGGATGTATCTTTTGTTAATT
GACGATACACACGATTTTTATCCAATGCACTGGTAACTGGTTTTGGTGATCCCTCTTGAATATTTAAAGGTACAACATCA
AGAATTGCAGTACCATTATCACGTAAATGATATTGCACAAGTGCACTATCTTTTGTTCTTGTCCATCCTTGGTCAAACAC
AAAGTTACCTAAACTATAGAAGATAATCCCTTGCTTATACACATCAAAAGATTGAAGTACATGCGGATGGTGTCCCACAA
TAATATCTGCCCCTGCATCAACCATTGCTTTTGCTAAGGCTTCCTGTCTAGGACTCGGTTTATTATCGTATTCTTCCCCC
CAGTGCGTATTTACTACGACAAGATCAGCATTACCTTTTTTAGGATCCTTTGCCTTACTAATTTGCTTAAGTAATACATC
TGGGTTCATACTTAACGAACCTGGTTGTTCTTTCGTTGCAATAGCTCCTGCTACAAATGCATCTGTAAATCCAAGAGTAG
CAACCCTAACACCATTTACATTTTGATACACAATATTTTTTACATCTTTGAAATTTTCACCAGCACCCACATAGTCAAGA
TCAGCTTCTTTAAAGGCCTTTATTGTATCTTTAGTTCCCTTAGCACCATAATCCGTCATATGGTTATTCGCCAAATTTAA
TACTGTAAATCCGGCTTCTTTTACTGCCTTAACTGTTTCTTCTTTTGCACTTAAGTGAATATTCTTATCTGCTTTTTGAT
AATTCTTTTTATCTTCTAACAAAACAGGATGTTCGAAATTCCCACTTACGTAATCTGAGTTTTTAAATATGGCGAAACA
TGACGAAAAACATAATCTGTACCGTAACGATTAACAATCTCTTTTACGTGACGTCCCATCATAATGTCACCAACCATCGT
CATCGTCAATTTTTCGTTCTCACGATGTTTTACTGGTGCTACTGCTTCTGTACGTTGTACCCATGTCGCAGCTATTAATA
TAACTGCGATAAGAGGTAATACGATTGCTACATAACGAGGATTTTTTTTCTTGGTTTTCTTAATAAAGATCAGTAACTTT
TCTTGAAATGTCAATTTTCGTCTCATTCTACCTCACCTTAAAATAAGTAATAAATATTCATGATTGCAAATGTTGCACCA
CTTAACAAAATTGTAGTTCCAATTGTTAATGGTAACCCTTGTCTTTGAATTGTATTTGCAATTAATCCTGGAACAATAAC
TCCAATACCACGGAATTCAAAAATCTCAAATGGCATAACAGGATAACAATAATCAAATAAAAGTTTTAAACAAATACCTG
TAATTAGCGTTGCCGCAAATTTTCTACGGCCATATAAAATCATGAATCTTGAAACACCATACGTAACGATTACATATGTT
AAAATACTGATAAATAAAACAACCAACATAAATACGGGCTGATTAAAAACGAGTGCTAAATAACCAGGTACAACTAAACC
TGCAGGTAAAATACCTGTTCTTTCTGTAAAAATAAGGCTCAGTGTAACTCCTAATACTAATGCAATATATAAATCTGATC
CAAACATTCCTGTCCCTCCACTTAAATCACTTTTGCTTGCTTTTTGCCAATTTGTTCTTCCATAATCATATCGATTAATG
GCTCAGCTGCACCATGAATATTACCCACTCCATATACAATCCGATTTTTTAAATATGGACGCATACGAGACATAATTTCA
CTTGTTGACCAGCCTTCTAAGTTCCAATACTCTTGCGTTGGAATATCTCCTTTTTCAAAAGCACTTGTAATAGGTGCAGT
CGTTTCTCCAATCGCAATAACTATTTCCGCTTTAATATATGGCAAAACATCCCTAGCAAACTGCTCAGTACGATCAACGC
GGTCAGGGCGGCAATTCATAATTACAATTGGAGCTAGATTACTATATCCAAAATCATCCACACGTTCCCAAATACGTAAT
GTTGATGAGGGATCATTCGCTGCAAAACCATTTACGAAGAACGCAGGCTTAGATTGGTCAGCAAAACGTGTAATTCTCAT
TGCTCCTGGATCCGGATGAGCATTCAACATACCACGGAATGCTGTTTCCTCATCAATCCCAAGAGCCTCTGCTACCGCTA
AAGCAAGCGATGCATTATCTGGGAAGACCATGTAATCAAATTTTCGTAAGAATTCTTCTGAAATTCTAGAATTATCCGCA
ACAATCACTTTTGTATTTCTCTCTTCTGCAACCTCTTTAAAGTAATCCAAGTATTCACTTTCAATAGTGACTAAATGTCC
ATTATATGGAATGGTAGCAGTGAAAGCTTCAGCT
>nmrc_008
TCTCCGATATTACGTCCTTGAAACTCACTCATCCCTACATCAGCAATCGCTTTTTCTACCTTTTCCTTATCGTTTTTCGT
GAAAAAGCGAAACAATCCTTTTTTCGAAACGAGTCCCATTGACACAACTTCAAACACAGTTGCCGGAAAACCAGAATTAA
AGCTATTTGCCTTTTGCGACACATAACCAACTTTGTTCCATTCTTTAAACTTCTTACTATCAACACCAAACAAACGAATG
CTTCCCTGTTTTGGCTTTAAAACGCCTAATAAACATTTTAGCAAAGTTGATTTCCCAGAACCATTCGGGCCAACTAAACC
TAAAAAAGCTCCCTTCGGAACTTGCAAATTAATATCTTCTAACACATTTCGATCTTCATATCGAAATGACAATCCTTCTA

TTTCTAATATATTATTCATAGCATCTCACCTATTTTAATTCAGAATGATTCCGATTTATATCTATTTGAATTATAGTACA
GCTTATTATAGTTGTAAACCAATCTGCCTAAAATATATCTACTTTTTCAAGAAACCTTACTATTATAACAAAAAGCAGAG
AAAAATTCTCTGCTTTCGAAAAATATTCCTATAAAATGAAACCTTAATGAGATGAGGCCATCCCCTACCGATTGGTAAAG
GCTTTTACTGGATAATTTTTTTAGTGAATAGCCGATTTAAACTTACCACCATTTGTCTCTTGTGTACTCATAATCGTAAC
AAATGCATTCGCATCAATTTCATACACAATTGATTTCAGCTTTGTCACTTCCAAACGCGTTACAACTGCATATATTACTT
CTTTTTCTTTATCCGTGTAACCGCCTTTCGCTACAAGCTTTGTAGTTCCACGACCAAGACGATGTAAAATCGCATTTGAT
ACTTCCTCATATTGATCTGATACAATTAAAACTGCTTTCGTTTCATCTAAACCTTGAATGACTGTATCAATTGTTTTGAA
CGCAATATAGTACGTCATAACAGAATACATAGCTTGTTCAACACCAAATACAAATGCTGCCCACGCAAAAATGAATAAGT
TCACAAACATTACAAATTCGCCAACGGAGAAAGGTAATTTTTTCGTTAATAAAATACCCATAATTTCTGTTCCGTCCATT
GATCCACCATGACGAATAACGAGCCCTACACCAAGCCCTAAAATAAGACCACCAAACACTGTCGCTAAAATTGGCTCTGT
TGTAAATGGCGGAACAGCATGTAACGTTGATTCAATAAACGCTAAAGCTACAATACCGAACGCCGAAGATAGCATGAACG
TTTTTCCTATTTGCTTATAACCTGAATACATAAATGGGATGTTGAGAATAACGACTAAAGTAGAGAAGCTTAACCACCAA
ATATTAGGAGTAAGATAATCTAGTATAAGAGAAATACCAATAATTCCACCGTCAATAATTTTATTCGGCATTAAAAATAA
TTCAATCGCTACCGCTGCACATGCTGCCCCAAAAATAATCATAACTAAACGATAAATAAGATGGATAACACTTTCTTTTC
GATGTTGCTTTTGCTCCATAAATCCTCCCCATACTCTTTTTCTATAGTTTTGTCTCTTTATTATAACATACGCTTCTTTT
TGTCATGAAAAAAGAAACTCTATACGAATATGTACAAGCTACGTACTCATATATTTCGATAGAAGAACGTGTGAAGGAGG
ATAAAACGATGAACCTCATTAAACAACTTGTAAATAAAAAATTAAATCATATTTCTACAAAAGATTTATTGAAATATAGT
AAAGAATATGAAGTTCCAATTACGACTGCACAAGCTGATCAAATTGTTGTACTTATGAAAGGAAAGAATATTAACATTTA
TGATAATGACGAACGACTAGAGCTCTTAAAACAAATAGCAAAAGTAACCTCCCCTGCTACTGCCCAACAAGTAAATACTT
TATTTCAGCAACTACTAAAATAAGGAGGGGATATCCCCTCCTTATTGCGCTTTAATTTTTTCAAGAAGTCCTTCATCAAA
AGTACCATTTTTCAGCATTTCGATTTCTAATTTATATGGCGGCTTCTTATCTTTTTTATCTTCACCTACATATGGCGTTT
CAAGAATTTTTGGTACGTGCGTTAACTGTGGATGATGTACAATATGATGCAATGCTTTATAACCGATATGACCGAAACCA
ATATTTTCATGACGGTCTTTTCCTGCGCCGCGTACATTTTTACTATCATTAATATGAAGTACTTGTAAACGATCGATACC
AACAATCTTATCAAATTCGTTTAATACACCGTCAAAGTTATTTACAATGTCATATCCTGCATCGTGCGTATGACATGTAT
CAAAGCATACTGATAGTTTTTCATTATATTTTACGCCATCAATAATTTTTGCAATTTCCTCGAAACTACGGCCGCATTCT
GTTCCTTTTCCTGCCATCGTTTCTAACGCAATGTTAACAGTCTGATCTGGCGTTAACACTTCATTAAGTCCTTTAATAAT
CTGTTGAATACCAGCATCCGCTCCTGCACCAACGTGCGCACCTGGGTGAAGAACGATTTGTTTCGCCACACCTAATGCTG
ATGTTCTCTCAATTTCCATACGAAGGAAATCTACACCTAATTGGAATGTTTCTGGCTTCGTCGTATTTCCGACATTAATA
ATATATGGCGCATGTACGATAATCTCTTCAATACCGTTTTGTTCCATATGTTTTCTTCCTGCTTCTATGTTCAATTCTTC
AATTGGTTTTCTTCTTGTATTTTGCGGTGCACCTGTATAAATCATAAACGTTGTTGCACCGTATGAAACAGCCTCTTCAC
TTGCTGCTAATAACATTTTCTTCCCGCTCATGGAAACATGAGATCCAATCTTTAACATACAATCACCTCTTCAATATACA
ATAGATATAATGATAGCATAATTTGTAGAAATATGTAGTGAATTGTTTGTTCACAAAAAACGCACTACATAAATTTATAC
GACTATTTAAACAGACTACACTCTTCATTTACATTATCTAGATGAAGCAAAACTTTAATCAATATTTTCTATCCATATTG
ATTAAAGCTCTCA
>nmrc_009
CAACCATACATGCAGCATCATCCCGAGCAAATGATCCCTCCTCAAATGTATGAATCAAACGAAACGCGCGGCGGTGCAGC
AACTACAGCAGCATCAAGTAGCGGCATCGGTAGTTTTTTTTCGAATTTAATTTCGAATCCAACTAATATGATAAATAATA
TCGAAAAAGTATCACAAGTCGTTCAATCTGTAAGCCCTGTCGTCGAACAGTACGGTCCCATTATGCGTAACCTACCAAGC
ATCGTTAAAATCCTCACCTCTGGAAAAAGTACGGAAGAAAATCCAACCGAAGATCAAACTGAAGACCTAACAGAAAAGGT
TGAAGTAGCAACTCCACCTCCTCCACAAAAAAAAAGAAAAAGAAAAAAAATGGTGATTGAGCCAGTTATAGAAAAAGAAGT
GCGCGAGGAGCCTGTTCAAAAAATAGCAACAAAACCAAAACTATATGTGTAACAATCCTTTGTTTTCTATCCACTCCTCC
TTTATAATGTAAAAGACTATGCACAAAAGTATCCCTTGTTTAGAAGGAGAGGATTACTCATATGAAAATTGTTAAAATTT
CCCCTCGTGGTTATTGCTACGGTGTTGTGGACGCGATGGTTATTGCACGTAACGCCGCATTAGATACATCATTACCAAGA
CCTATTTATATTTTAGGTATGATTGTTCACAACAAACATGTAACAGATGCATTCGAAGAAGATGGTATCATTACATTAGA
CGGTCCAAGTCGATTAGACATTTTAGATAAAATCGATTCTGGTACTGTTATTTTCACTGCACACGGTGTTTCTCCAGAAG
TTAAACAGCGTGCAAAAGAAAAAGGTTTAACAACCATCGATGCCACTTGCCCAGATGTTACAAAAACACATGACCTTATT
GAAGCAAAGAAAGCTGAAGGTTACCATGTCATTTATATCGGCAAAAAAAATCATCCAGAACCGGAAGGCGCAGTTGGGAT
CGCACCTGATATCGTTCATCTTATCGAAAGAGCCGATGATTTAAAAACATTAGAAATTCCAACGGATAAAATTTTAGTTA
CAAATCAAACAACGATGAGTCAATGGGATGTGCAACATTTAATGGAGGACATTCAGAAAAAATTCCCAACAGCAGAGTTC
CATAAGGAAATTTGTTTAGCAACTCAAGTTCGCCAAGAAGCTGTTGCCAAACAAGCTGATGTTGCCGACTTAACAATTGT
TGTTGGTGATCCGAAAAGTAACAACTCAAACCGTTTAGCACAAGTATCACAAGAAATCGCTGGTACGAAGGCATACCGCG
TTGCAGACGTAAGTGAGATTAAATTAGAATGGCTACAGGGTGTAGAAAACGTAGCTGTTACAGCAGGTGCTTCTACTCCA
ACACCAATTACAAAAGAGGTTATCGCTTTCTTAGAGCAATATGACCCAATGAATCCCGCTACATGGGAGAGAGTTCGAAA
AGTACCGTTACAAAAAATATTACCTCGTGTAAAAGTGAAAAAAGAACAATAATAAAAACCGTTGCCTATATGAGCAACGG
TTTTTATTTTTCTTATACAAATGTAAATGGATCTGTATGTAACTGCGAAGCATGAATGTTTACATTAAGTTTCTTTGCAT
CCACTTTTTCTTGTAATTGCTTTTGTACACCTTGCTTCATTACCTTTTCAACGTTATGTCCTGGGTCAACTATATTTAAA
CCGAGCATCATCGCATCATGAGCAACATGATAATACATGTCCCCCGTTACATATACATCTGCTCCTTTAAATTTAGCTTG
ATTGATGTATTTGTTACCATCGCCACCAAGTACAGCTACTTTGCGCACTTTATCATCTAATTTCCCAACAACTCGCGCAC
CCTTTACATCTAATGACTTCTTTACATGTTCCGCAAATTGTCCAAGTGTCATTTCTTCTTGTAAATATCCTATTTTTCCA
AGCCCTAATGTTTCACCTTTGTTATCAAGTGGATACACATCATATGCTACTTCTTCATATGGATGTGCCGTTACCATTGC

TTTAATTACTTTTCGCTGTAATGAAGCTGGAATAATCGTTTCGATTCGCACTTCTTCCACGCGTTCTAACTGCCCAGTTT
CCCCGATATAAGGATTTGTTCCCTCT
>nmrc_010
TTTCATTTGCGCAAAAAAATGTACGTCAGGGAGGGAAGGACGCTCGTCCTAAATGAATGGAAAAAAGTTGGTATGTTGTC
CATACTTATTCTGGATATGAAAATAAAGTAAAAGCAAACCTAGAGAAACGTGTAGAATCAATGGGTATGCAAGATAAAAT
TTTCCGTGTTGTTGTCCCAGAAGAAGTAGAAGTAGAAATGAAAAACGGTAAAGAAAAATTAATGAAAAGAAAAGTGTTCC
CAGGTTATGTATTAGTAGAATTAATCATGACTGATGACTCTTGGTATGTTGTACGTAACACGCCGGGTGTAACTGGGTTC
GTTGGCTCTTCTGGTTCTGGATCTAAACCATCACCTCTATTAGAAGAGGAAGTTGTTACCATTATGAAACATATGGGAAT
GGACAACGAAGTGGTTGATTTCGACTTTGAACTTCATGAGACAGTACGTGTAAATGAGGGACCATTCGCAGATTATACAG
GTGCTATCGAAGAAATTGATGTGGAGAAGAAGAAGGTTAGCGTACTTGTGGACATGTTTGGTCGCGAGACTCCAGTTGAA
CTTGACTTCCATCAAATTGAAAAATTATAAAATGAAACTTGAAATGAATTGTAAAAAGTGATAATATCTTTTAAGTCAGT
ACGTCTTCGTTATCGGAGACGTTTTTTGAAAGATTTTATCCTTACAGATAAAATATGACGTGGGAGGGCAAATCACTGTC
CAATTGACCACATCACGGACTTAAGGAGGTGTGTCTCGTGGCTAAAAAGGTAATTAAAATGGTAAAACTTCAAATTCCTG
CAGGTAAAGCTAACCCAGCTCCACCAGTTGGTCCAGCATTAGGACAAGCAGGTGTTAACATCATGGGCTTCTGTAAAGAG
TTTAACGCTCGTACAGCAGATCAAGCTGGTCTTATCATCCCTGTTGAAATTACGGTATTTGAGGACCGTTCATTCACTTT
CATTACTAAAACTCCTCCTGCTGCTGTTCTTCTTAAGAAAGTAGCTGGTATTGAGTCTGGTTCTGGTGAACCAAACCGTA
ATAAAGTGGCAACTGTTAAGCGTGATAAAGTACGCGAAATCGCTGAAACTAAAATGCCTGACCTAAACGCTGCTAGCGTA
GAAGCTGCAATGCGTATGGTTGAAGGTACTGCACGCAGTATGGGCATCGTTATCGAAGACTAATTCGATTTGTTTTTTAA
AAAAGGTTGCGGGTCTGGAATTCCAATTCGCAACCTTTATTATCGTAAATGATTATCGTTTTTAAATAAATGGATGGCGC
GCATCCTCAGGTTATACCTGAAAATAAGCGTAAACGTGGGAGGTTATTCCGCTAAAACCACATTCGAGGAGGAAATAAAA
ATGGCTAAAAGAGGTAAAAAGTACGTAGAAGCTGCAAAGCTTGTTGATCGTGCAGCTGCTTACTCTGCAACAGAAGCAGT
AGAATTAGTAAAGAAAACAAACACAGCTAAATTTGATGCAACTGTAGAAGCTGCATTCCGTTTAGGTGTTGACCCTAAGA
AAGCTGACCAACAAATCCGTGGTGCAGTTGTTCTTCCACACGGTACTGGTAAAGTACAACGTGTATTAGTATTCGCTAAA
GGTGAAAAAGCTAAAGAAGCTGAAGCTGCTGGAGCTGACTTCGTAGGCGATGCTGATTACATCGGTAAAATCCAACAAGG
TTGGTTCGATTTCGATGTAGTAGTAGCAACTCCTGACATGATGGGTGAAGTTGGTAAACTTGGTCGCGTATTAGGACCTA
AAGGTTTAATGCCAAACCCTAAAACTGGAACAGTTACTTTCGATGTAACTAAAGCTGTTAACGAAATCAAAGCTGGTAAA
GTTGAATACCGCGTTGATAAAGCTGGTAACATCCACGTTCCAATCGGTAAAGTATCTTTCGAAGATGCTAAATTAGTAGA
AAACTTCAGAACAATTGCTGACACTTTACAAAAAGTTAAGCCAGCTGCTGCAAAAGGTACTTACATGAAGAACGTAACAG
TTGCTTCTACAATGGGACCTGGCGTACGTGTAGACGTTTCTACATTAGCGTAAATTTTGGAAGTTGACTTCATAAAGAAG
TTTTTATATAATCATTTATGTTGTGAATTTAAATAGTGTACCGTAGACAGTAGGTGTCAATAGACTTAATTTCCTACCTA
GGTGTTAATATACGAAGCGGAATTTTTTTCTGTGACTATATGCCTCCATGTCTACAAGTTGGGCATGGAGGTTTTTAGTG
CACTTTCGGTACATCTTCTATATAATCTACAGGAGGTGTAATAACATGAGCAAAGTAATCGAAACTAAACAACAAGTTGT
AACTGAAATCGCGGACAAACTTCGCGCTAGTAAATCTACAATCGTTGTTGACTACCGTGGTTTAACAGTTTCTGAAGCAA
CAGAATTACGTAAGCAATTACGTGAAGCTGGCGTTGAGTTCAAAGTTTACAAAAACTCTCTAACTCGTCGTGCTGCAGAA
TCTGCTGAAATGGCTGAGTTAAATGAATTCTTAACAGGACCAAACGCAATCGCGTTCAGTAACGAGGATGTAGTTGCTCC
TGCGAAAGTATTAAACGACTTCGCTAAAGATCATGAAGCTTTAGAAATTAAAGCGGGCGTAATCGAAGGTAAACTTGTAA
CACTTGATGAGGTTAAAGCTATCGCTACTCTTCCATCACGTGAAGGCTTACTTTCTATGCTTCTTAGCGTTCTTCAAGCT
CCAATCCGTAACCTTGCACTTGCTACTAAAGCAGTTGCAGACCAAAAGGAAGAGCAAGGCGCTTAATTTTTTAAAAGATA
ATTACGTATTATCGATAAAACAATACAAACCTATTTAAGGGAGGATATTTACAATGACTAAAGAACAAATCATTGAAGCA
GTTAAATCTATGACTGTATTAGAACTTAACGACTTAGTAAAAGCTATCGAGGAAGAATTCGGCGTAACTGCTGCTGCTCC
TGTAGCTGTTGCTGGTGGCGCTGGAGAAGCTGCTGCTGAGAAAACTGAATTTGATGTGGAACTAACTAGCGCTGGTGCAC
AAAAAATCAAAGTTATCAAAGTTGTTCGTGAAATCACTGGTCTTGGCTTAAAAGAAGCTAAAGAATTAGTTGACAACACT
CCAAAAGTAATCAAAGAAGCTGCTGCTAAAGAAGAAGCTGAAGAAATCAAAGCTAAACTTGAAGAAGTTGGCGCTGCTGT
AGAAGTTAAGTAATTAACTTTTGATGCTTTAAAAAAAGCTCGCTCTCATGCGAGCTTTTTTTAACTGTAAAGAAAAGAGG
TGGCCATATGGCAGACCATTATTTTTCTAACGACCCTTCTAGTAAAAGTGATCGTAAGCGATGGGAATTTACGCTTCGTG
GATCTCGATTTACTTTCTTATCTGACCGTGGGGTGTTCTCGAAAAACGAAGTGGACTTTGGTTCTCGTCTTTTAATTGAA
GCGTTTCAAGTGCCAGATATTAAAGGTGATATATTAGACGTAGGTTGTGGATATGGACCGATTGGTTTATCGTTGGCGAA
AGAGTTTCAAGACCGTAAAGTTCACATGGTGGATGTGAATGAAAGGGCGCTTGAGCTTGCGAAAGAAAATGCCGCTAACA
ATAGAATTGGAAATGTGCACATTTTTCAAAGTAGCGTCTACGAAAACGTAGATGGTATGTATGCTGCTATTCTATCTAAT
CCTCCAATTCGTGCAGGTAAAGATATCGTGCATGAGATTTTAGAAAAAGCTGTAGAGCATTTAGTTCCAGGTGGAGAGTT
GTGGATTGTTATTCAAAAGAAACAAGGTGCACCATCTGCACTGAAGAAACTAGAAGAAGTGTTTTCTGAAGTCGAAGTTG
TAGAAAAGAAAAAAGGATATTATATCATAAAATCAAAAAAACGTTGACGGTTATTTTTGGCTATGTTAACATTATACAAT
GCCAATATATGATTTTCTGCGTTGAGAAAGATGTATATTTTTGTTTCTCTTGGAAAAGATAGTAAAATCAGCAGATTATG
AAACAGAATGATGGTTTTCTTATAGAAGCCATTTTTCTTTTTTGAGCAGGTAGAAAGACTCAACGTATTTATCTTTAAGA
GAAAAAAGACTACGCTAAATAGCAGTAGTTGTATTTATTTGTGATTTTGCACAAATTTTTTGTGCATTTATAATACTCAT
GATTTGAGGGGTGAAGCAGTTGACAGGTCAACTAGTTCAATACGGACGCCACCGCCAACGAAGAAGTTATGCCCGTATTA
GTGAAGTATTAGAGTTACCAAATCTTATCGAAATTCAAACCTCTTCTTATCAGTGGTTTCTTGATGAGGGTTTGCGAGAA
ATGTTCCAAGACATTTCTCCGATTGAAGACTTTACGGGAAATCTATCGCTTGAATTTATCGACTACAGCTTAGGTGAACC
TAAATACTCTGTAGACGAATGCAAAGAGCGTGATGTGACGTATGCAGCACCACTTCGTGTAAAAGTGCGTCTAATCAACA
AGGAAACTGGTGAAGTAAAAGAACAAGATGTGTTCATGGGAGATTTCCCACTCATGACAGAGACTGGAACATTCGTAATT

AACGGTGCAGAACGTGTTATCGTTTCCCAGTTAGTTCGCTCTCCAAGCGTATACTATAGTGGCAAAGTGGATAAAAACGG
AAAACGTGGTTTTACTGCTACTGTAATTCCAAACCGCGGAGCTTGGTTAGAGTATGAGACAGATGCTAAGGATGTTGTAT
ATGTGCGTATTGACCGTACGCGTAAACTTCCTGTAACTGTTTTGTTACGCGCATTAGGGTTTGGCTCTGATCAAGAAATC
ACCGAGCTTTTAGGTGATAACGAATACTTAAGCAACACATTAGAAAAAGACAACACAGATAGCACAGAAAAAGCATTGCT
TGAAATTTATGAGCGTCTACGTCCTGGTGAACCACCAACAGTAGAAAATGCCAAGAGCTTACTTGTGTCTCGTTTCTTCG
ATCCAAAGCGCTATGATTTAGCAAATGTAGGTCGCTACAAGATCAACAAGAAGTTACACATTAAAAACAGATTGTTTAAC
CAACGTTTAGCTGAAACATTAGTAGATCCAGAAACTGGTGAAATTTTAGCGGCAGAAGGAACAATCTTAGATCGTCGTAC
ACTTGATCGCATTTTACCTTACTTAGAGAAAAACATTGGATTCAAAACAGCGAAACCAATGGGTGGAGTGGTAGAAGGTG
ATGTTGAGCTGCAATCTATTAAGATTTATGCTCCTGAGTCAGAAGGCGAGCGCGTAATTAATGTAATTGGTAATGCAAAC
ATTACTCGTGATGTAAAACACATCACACCAGGTGATATCCTTGCTTCTATCAGTTACTTCTTCAACTTACTATACAAAGT
AGGGGATACAGATGATATCGACCACTTAGGAAACCGTCGTCTGCGTTCTGTTGGAGAACTATTACAAAATCAATTCCGTA
TCGGTCTTTCTCGTATGGAACGTGTTGTTCGTGAGAGAATGTCGATCCAAGATACAAATGCAATTACACCACAGGCATTA
ATTAATATTCGTCCTGTTATTGCAGCTATTAAAGAGTTCTTCGGAAGTTCTCAGTTATCTCAGTTCATGGACCAAACAAA
TCCATTAGCAGAGTTAACTCACAAACGAAGATTATCTGCATTAGGACCTGGTGGTTTAACGCGTGAGCGCGCAGGCTTTG
AAGTACGTGACGTTCACTACTCTCACTATGGTCGTATGTGTCCGATTGAAACACCAGAGGGACCAAACATCGGTTTGATT
AACTCATTATCTTCGTTCGCGAAAGTAAATGAGTTTGGTTTCATTGAAACACCATACCGTCGTGTTGACCCAGAAACTGG
TCTTGTAACAGGGCATGTTGATTATTTAACAGCAGATGAAGAAGATAATTATGTTGTAGCCCAAGCGAATATGAAATTAT
CTGAAGAAGGAGAATTCCTTGATGAAGATATCGTAGCTCGTTTCCGTGGTGAAAACATTGTCACAAATAAAGAACGCATC
GACTACATGGATGTATCACCAAAACAAGTAGTGTCGGCAGCGACAGCTTGTATTCCGTTCTTAGAAAACGATGACTC
>nmrc_011
TTATAGGCTCTGGAGCTGTAATGTTGAGCGGGCCATCAATTTCTTTTTTGTGTATGATGAAATCAATCATGGAAGCGACA
TCGTCTATATGAATCCATGATAACCATTGCTTTCCAGATCCAACTGTACCTCCGATATAGAATTGATAGGGAAGTAGCAT
TTTGGGAAGGGCTCCTCCGTCTGGACCTAATATGACTCCGAATCGTGCATAGATTGTTCGTATTCCGAGAGAGCGTGCTT
TAGATGCTTCTTGTTCCCATGAATATACTGTATTTGCTAAAAAGTCATTTCCGGGAGTCACATGCTGTTCTGTAAAAGAC
TCGGTTTCAGACGTTCCATAGTATCCAATTGCACTCGCGTTAATAAATGTATGTGGTTTTGCAGGGAGTGCCTGCAATTG
TTTAATGAGTCCTTTTGTTGTTTGAATTCTACTGTTTAAAATGATTTTCTTTTGTTTCTTTGTCCATCTACTATTAATAG
ACTCTCCAGCTAGATTAATAACTACATCAATAGAAGAGAGAGGGAAGGTTTGTAAATCTGGTGTCCATTGCACATACTGA
AGGTTAGGGTGAGAAGTTTCAGTAGTTTTTTTCTTGTGAGAATGTAAACCGTATATCCTTTTTGAATAAAAAAAGTAGA
AAGGTATGTGCCGATAAAGCCGGTACCACCAGAAATTGCGATTTTCACAATTATTTCCTCCTCATTATATATATTCTTGA
AAAGCAAAAACGTTCCTCGACTATGTTAAAATAATAGAGAGGTGAGAGTATGGCTGTCATTACAAAAATAGAAGTGCAAA
AACGATCGAAAGAACGGTTTAATATTTATATCGATAAAGGTCAAGGTGAAGAATACGGATTTAGTGTGAACGAAGTAATC
TTAATAAAGCACGGATTACAAAAGGGCTTAGAAATTGATGAAATAGCGTTAGGAAATATTTTGTACAATGAAGAGGTACA
AAAAGCATATTTACAAGCAATCTCCTATTTATCCTATCAAATGAGAACGAAACTAGAAATAGAAGATTTCTTACGAAAAA
AAGAAGTGGGACAAGCCATCATCTCTGAAGTCGTTTCGAAATTATTACATGACCGATATATTAATGATAAAGAGTATGCT
ATTTTATATACGAGAACGCAAAGTAATGTGAATCGAAAAGGTCCAACGGTTATTAAAAGAGAGTTGTTAAATAAAGGTGT
TCAGGATCTAATTATTATGCATAGTTTACAAGAATATACGAAGGAAAAGCAAATTGAGAATGCTTTAATTCTTATAGAAA
AGAAGAAAAAGTCTTATCAAAAGCATTCCTTTTTACAAATGAAACTAAAGTTAGATGAAATGCTTGTTCGTAAAGGATAT
TCTAGAGATGTGATTCAAATTTGTTTGGAAGAATTGAAAGACGAAAAAGATGACGAAAAGCAACAAGAAGCGTTACACTA
TCATGGGAACAAATACTATGAGAAATATAAGAAGTATGATGGATGGACGTTTGAAAATAAGATGAAACAAGCGTTATATC
GTAAAGGATTCTCTATTGATGAGATAGAGATATTTTTGCAAATGAAACGTGAAGAGGGATGAGGGAATAAAATGAATATG
CCAAAACGCTACAGTGAAATGACACCATATGAGCTAAGGGAAGAAATTGGGGTTTTGAAAGAGCAAGCAATAAAAGCTGA
GCAACTTGGAATTGTAAATGAGTTCGATGTATTAATGCGAAAGATAGCAATGGCTCGTGCTTATATGACTGACATAAATA
AATTCCATATTGGTGAGACGTATGAATTAGTAGAAGAACCTGGTATATTATTTGAAATTACGTATTTCAATGGGGTATTT
GCTTGGGGACATAAGCAAAATGATAATGAAGAGATTGGAATACCGATTTCTTTATTACAAGAAAAATAAAAACCAGAGAG
CGAATATCTCTCTGGTTTTCATCTTTGGTAGGCGTTACAGGAGATAGGAGATTCTAAAATTGGCGTTTTTTACATTCATT
CGCCAATATAATGAGAGACTGGGTTTGTTGCGTCTGTCCGTTAACTTGCGCTTTTGCATGTTTCGGACGAGTCCACGTAT
GGTTAAATAATTCAGAACGACTATCTAAGCGATCACGGTAGCTCATCTTTATCACCTCGTGTAGGAAGTTGAAGTTAAAT
TACTTTGTACTACTCTTCCTGCTGATTTGCAGCACGCATACGTTCTTGTGGGTGCGTGTTAATTGTGCCGTTAGGTCGCT
TCGAAGCGAAGCGTGATTTCGCTTTCGGTTGACCGTCGATTTTGCTGTTGTTTTTTGACTCAGACCAAAATTCGGCTTGT
TTACCCATTGCGAACGCCCTCCTCATCATCAGTTGATACCTCTTGAAGAAGTATCAATTATAGAATGTGCAAAATAGAAG
AAACTATCCTTTAGAAATGAAGGGATAATTAGGTAAAGGAGATTAGAACATGAATGATATTTATGAAGCATTAACGAAAG
AACTATTAGAGAAAAATGACAAACTTTCTTATGCACAAGCTCGTGCGTGGGTTGAATTACTGTGGGAAGATTTTCAAACA
ACTTATGCGAAATCAGGTCGTTATCAAGGAGAAGAAATGACTGAACAAGTGGTGCGATCATGGATTAATAATCATGGAGG
ACGCCTTCATGAAATGCGTACAAATAATCCGAAATATAGCCATCTAATCAATCAAGAAGATCATTTGAA
>nmrc_012
GGAAGTAACTCCAGTTTTTTTCGAAGTTTTTCTTCACTAAAAATCCATCCTGTATAGGAACTAATAATATTTAAATTATG
ATCGAGTTGAACGATTGCAACGAATGGATAATAATCTTTACTGCGATATCGCAAATCAATGAATCGAACTTCATAATAAT
GATCATAATCGAAAATATCCCAGCGATACACAGGAGAGAAAGATAAAAACGCTGAGATGTTTTCATCTTGTTTAGCTGCG
CGCATAATAGCATTATCTGGGAACGGTACCCGATCGAACTTGTCATATATCATGATATTACCACGGTGCCATCTCGCAAC
GTAGTAATAATCAGCCGTAACGACTGCTAAATGGTAATGATAAAATCGGTAAGAAGGAGAAATGATAATCTTTTCGACAT

TTTTGAAACGTTTGTGTACAACGCTTCTTATATTTCTGTGCATAATAATCCGTCCGATGTAGTAAATAATCATTAATATA
TACGCTGCTAAGGCAGTATACCCTTTATGGGAACCTACGAGCATACAAGCAATTGCGAGTATATGGATAAAGAAAATGAC
GGTATCAAATGTATTAATGATGCCGAGTGCTACCCATTTTTTTGTGAAGGGCCGTAATGCTTGTGTACCGTAGGCATTGA
AAATATCTACAAAGACGTGAAGAAAGACCGCAATAAATGACCAAAGTAGTAGATGAAGATATGGAGCGTCTGAAAAGAAG
GCGAAAGAGATGCCACTTATAAGGAACGACCAAAGAATTACTGCAGGAATGGAATGAGTAATCCCGCGATGATTTCTTAT
ATATTTAGCGTTATTACGCAATTTTAAAACTGTATCGATGTCAGGAATATTGGAACCAGCAATTGTAGCAAGCATAACAG
CTTGAGGACCAATATCACTTTGTGCTATGGCTGGATCTAACGTGGCCAAACTGCCTAGAGTAACGCCCATAACAAGGTGA
GTGGCTGTGTCCATAGAAATCCACCTCCGTTTTTTATTAATGTAACTCTTTTTATTCGATACCTCAAGTCCTTTGCCTTC
TATTTCCTATGTCATCTTACTTAAATTTTTTCCTAAAATCTTTATAATAGTACTTATATGCAAAAAAATGAGGGGGATCA
AGTTTGACACTTGAAATATTAAATAACTTTAACATAGAGCAGTTTCAAAATGATTTAATTGGTTGGTTTGAAAAAGAGCA
ACGTGACTTACCGTGGCGTAAAAATAAAGATCCATACCGCGTTTGGGTTTCGGAAATTATGTTGCAGCAAACGAGGGTAG
AAGCTGTAAAACCATATTACGCGAATTTTATGGGGAAGTTTCCTACTCTAGAAGCTTTGGCCAATGCTGATGATGAAGAA
GTGTTAAAAGCATGGGAAGGTTTAGGGTATTATTCTAGAGCGCGAAATTTACATGCGGCTGTAAAAGAAGTAAAAGAAGT
ATATGGCGGGATTGTGCCAAGTGATGTAAAGAAGATTGAGAAATTAAAAGGAGTCGGCCCCTATACAAAAGGTGCTATTT
TAAGTATCGCATATGGCATACCAGAGCCGGCAGTTGACGGAAATGTTGTGCGTGTATTGTCTCGTATTTTATCAGTATGG
GATGACATTGCAAAACCGAAAACTAGAAAAGTGTTTGAAGAGATTGTGCGTGAAATTATTTCGGCTGAAAATCCATCTTA
TTTTAATCAAGGTTTGATGGAATTAGGAGCACTGATTTGTATTCCTAAAAATCCAGCATGCTTACTTTGTCCTGTTCGTG
AGCATTGCAGGGGATATGCTGAAGGTGTTCAAAAGGAATTACCAGTAAAAAGTAAAGCGAAAGCTCCTACGATGGTACCG
ATTGTTGCAGGAGTACTTCAAACGGAAGATGGTCGTTACGTCATTAATAAACGTCCAAGTACCGGTTTATTAGCTAATAT
GTGGGAGTTTCCGAATGTTGAACTTGGCGAAGGGATTCGTAATCAGAAGGAACAGCTTATAGATTATATGAAGGAAAAAT
TTGAGCTTTCGATTTCTATTGAAGAATACGCAATGAATGTACAACATACGTTTACACATCGTACTTGGGATATATTCGTA
TTTTATGGAAAAGTAACTGGTGATATTGTTGAAACAGATACATTGAAATTTGTATCGAAAGAAGCATTTGAGCAGTTACC
TTTCTCTAAATCGCATCGTACGATTTATGAGAATTGTGTTGAGAAAATTACAATGCAATAAACAAAAAAAGTGTTCCCAG
GTTGGGAACACTTTTTTAATCGTAAGAAATTTTTGTCCCATGAGTCCAACTAGCTTCACCTTCTTGTAAGCCACCTCTTG
ATTCAATTTCTATTACAATTTCTTTGTAAATACTTTGACCTTCCGCGTTTAAATAAGGCAAAATCTGTTGTAATGAATGA
TGAAAATAAGCTAATTCATCTTCTTTCCATTCGTTTTTTGAAACCATTGTTAGTTCAGTCATATCGCGTCCTATATACAT
ATTCCCACCTCCATTTTCTATAGTTTGAATGAGTAACAAGAGATATATGCGGAATTTTTTCAAAAGAAAATTACGGATAG
CTTCTAGTTGAATTGGTTACATTATTGATTGTGGAGGTGAGAAAGATGAGTAAAAAACAACAAGGTTATAACAAGGCAA
